# A neuroprotective astrocyte state is induced by neuronal signal EphB1 but fails in ALS models

**DOI:** 10.1038/s41467-017-01283-z

**Published:** 2017-10-27

**Authors:** Giulia E. Tyzack, Claire E. Hall, Christopher R. Sibley, Tomasz Cymes, Serhiy Forostyak, Giulia Carlino, Ione F. Meyer, Giampietro Schiavo, Su-Chun Zhang, George M. Gibbons, Jia Newcombe, Rickie Patani, András Lakatos

**Affiliations:** 1 John van Geest Centre for Brain Repair, Department of Clinical Neurosciences, University of Cambridge, E.D. Adrian Building, Forvie Site, Robinson Way, Cambridge, CB2 0PY UK; 2 Department of Molecular Neuroscience, UCL Institute of Neurology, University College London, London, WC1N 3BG UK; 3 Division of Brain Sciences, Imperial College London, Burlington Danes Building Du Cane Road, London, W12 0NN UK; 4 Institute of Experimental Medicine ASCR and Charles University in Prague, Department of Neuroscience, Videnská 1083, Prague 4, 142 20 Czech Republic; 5 Sobell Department of Motor Neuroscience & Movement Disorders, UCL Institute of Neurology, University College London, London, WC1N 3BG UK; 6 UK Dementia Research Institute at UCL, UCL Institute of Neurology, University College London, London, WC1N 3BG UK; 7 Waisman Center, University of Wisconsin, 1500 Highland Avenue, Madison, WI 53705 USA; 8 Department of Neuroinflammation, UCL Institute of Neurology, University College London, London, WC1N 1PJ UK; 9 The Francis Crick Institute, 1 Midland Road, London, NW1 1AT UK; 10 Addenbrooke’s Hospital, Cambridge University Hospitals, Hills Road, Cambridge, CB2 0QQ UK

## Abstract

Astrocyte responses to neuronal injury may be beneficial or detrimental to neuronal recovery, but the mechanisms that determine these different responses are poorly understood. Here we show that ephrin type-B receptor 1 (EphB1) is upregulated in injured motor neurons, which in turn can activate astrocytes through ephrin-B1-mediated stimulation of signal transducer and activator of transcription-3 (STAT3). Transcriptional analysis shows that EphB1 induces a protective and anti-inflammatory signature in astrocytes, partially linked to the STAT3 network. This is distinct from the response evoked by interleukin (IL)-6 that is known to induce both pro inflammatory and anti-inflammatory processes. Finally, we demonstrate that the EphB1–ephrin-B1 pathway is disrupted in human stem cell derived astrocyte and mouse models of amyotrophic lateral sclerosis (ALS). Our work identifies an early neuronal help-me signal that activates a neuroprotective astrocytic response, which fails in ALS, and therefore represents an attractive therapeutic target.

## Abstract (web summary)

Astrocytes can have protective or detrimental effects on neurons during injury, but the molecular mechanisms that determine these different states are unresolved. Here the authors identify a pathway via neuronal EphB1 that induces neuroprotective signalling in astrocytes through ephrin-B1 mediated STAT3 activation, which is impaired in models of amyotrophic lateral sclerosis.

## Introduction

Activated astroctyes can support motor neuron survival and recovery of their synaptic input following moderate neuronal damage^1^. Astrocyte inflammatory signalling through STAT3 plays a crucial role in these repair mechanisms, and is a hallmark of the protective astrocyte phenotye^1–4^. Elucidating mechanisms underlying this beneficial motor neuron-to-astrocyte signalling may therefore provide novel therapeutic targets for neuroprotection. This is of important relevance to amyotrophic lateral sclerosis (ALS), a devastating and invariably fatal disease leading to progressive degeneration of motor neurons in particular. Specifically, the breakdown of neuron-glia communication or a toxic gain of function have been demonstrated to play pivotal roles in neuronal dysfunction and death^5–7^.

Canonical astrocyte activation involves a cascade of cytokine signalling and inflammation following somal or axonal damage to neurons^8, 9^. Putative triggers include the release of various cytokines, such as IL-6, by injured motor neurons and inflammatory cells or, at later stages, cellular debris^10–14^. These responses shift the position of astrocytes along an inflammatory spectrum^8, 15^, determining if the astrocyte is in a protective or deleterious reactive state. The early signalling events responsible for regulating astrocyte reactivity in this manner remain poorly understood. A group of Ephrin receptors (Ephs) and their ephrin ligands are possible mediators of this regulation, given that they are crucial in bidirectional neuron-glia communication and undergo expression changes following injury, during plasticity^16–20^ and in neurodegeneration^21, 22^.

Astrocyte reactivity with a proinflammatory transcriptional and translational profile characterises superoxide dismutase1 (SOD1)-mutant mouse ALS models^23–25^. This is a relevant pathological aspect as glial inflammatory processes may exacerbate motor neuron dysfunction in ALS^15, 26–28^. However, astrocyte-mediated restorative STAT3-dependent mechanisms have also been demonstrated to occur in the context of traumatic motor neuron injury-associated inflammation^1^. Whether this restorative STAT3-mediated astrocytic activity is suppressed in ALS has not been directly addressed. There is some evidence of astrocytic STAT3 activation in the SOD1 ALS mouse model^29^. However, the degree to which this reflects a compensatory protective profile remains unclear. The detailed investigation of astrocyte-mediated pathology to directly address this issue has been hampered by the lack of appropriate human ALS model systems^30^.

Here, we have addressed whether Eph–ephrin signalling could serve as a primary neuronal injury cue invoking a potential protective astrocyte phenotype, and whether this response is altered in human ALS patient-specific astrocytes. Our study focussed on EphB1 evoked response, because this particular Eph is strongly upregulated in injured neurones^17^. We also investigated IL-6-induced signalling, which has been associated in a context-specific manner with both a deleterious proinflammatory and protective anti-inflammatory profile^31^. By integrating mouse in vivo and in vitro models with human iPSC-derived astrocytes, we provide direct evidence that EphB1 can induce early astrocytic STAT3 activation via ephrin-B1 signalling. This signal transduction pathway promotes a protective transcriptional profile, distinct from that seen for IL-6. Using patient-specific iPSC-derived astrocytes we show that this EphB1-induced pathway is impaired in SOD1-mutant astrocytes compared to their control counterparts. This study reveals a failure of astrocyte plasticity, reflecting a novel and potentially therapeutically targetable non-cell autonomous disease mechanism in ALS.

## Results

### EphB1 is upregulated in axotomised motor neurons in vivo

**Fig. 1:**
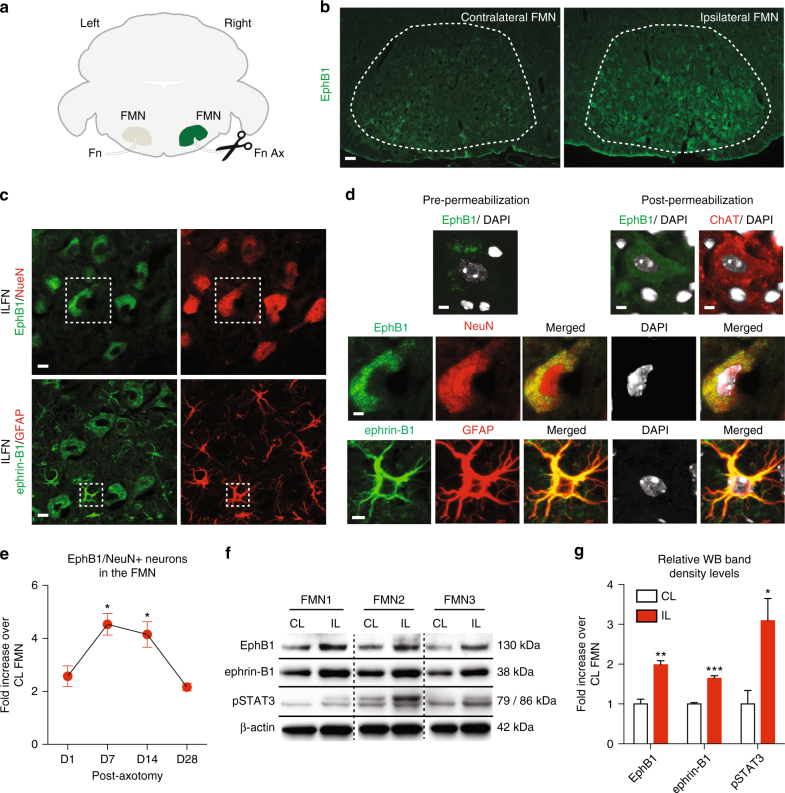
EphB1 is upregulated in axotomised neurons, where astrocytes display ephrin-B1 IR. **a** Schematic diagram demonstrating right-sided unilateral facial nerve (Fn) axotomy (Ax) and the associated ipsilateral (IL) facial motor nucleus (FMN) in which immunofluorescence was analysed in comparison with the non-axotomised contralateral (CL) FMN. **b** Representative immunofluorescence images showing abundant EphB1 IR 14 days after axotomy in the IL FMN in which 98% of neurons are defined as motor neurons^34^. **c** Images showing EphB1 and NeuN labelling and also ephrin-B1 and GFAP IR in corresponding neurons or astrocytes (ACs), respectively. **d** High magnification images demonstrate cell surface labelling for EphB1 in sections immunostained before permeabilisation and also cytoplasmic EphB1 IR post-permeabilisation in a ChAT-positive motor neuron (MN) (see also Supplementary Fig. 1) and NeuN positive neurons with motor neuron morphology^32^ in the middle panel. In the lower panel ephrin-B1 positivity is demonstrated in GFAP-labelled astrocyte soma and processes (see also Supplementary Fig. 1). **e** Graph showing the mean of fold changes in the proportion of EphB1-positive neurons in the IL FMN normalised to the CL FMN following axotomy (1, 7, 14, 28 days; *n* = 3 per time point, respectively, and 3–4 brainstem sections in each group; *p = 0.016, *p = 0.046 compared to values at day 1, F = 9.431; one-way ANOVA with Dunnett’s post hoc test; see also Supplementary Fig. 1). **f** Western blot (WB) showing EphB1, ephrin-B1 and pSTAT3 protein levels (both 79 and 86 kDa isoforms) in tissue lysates from both the IL and CL FMN of three WT mice at day 14 post-axotomy (see also Supplementary Fig. 8). **g** Bar graph represent relative WB band densities of EphB1, ephrin-B1 and pSTAT3 in the IL FMN and is expressed as fold increase over the band density levels for the CL FMN after normalisation to β-actin (*n* = 3 mice, unpaired *t*-test, **p = 0.006, ***p = 0.0004, *p = 0.034). Data presented as mean ± SEM. Scale bars: 50 μm for (**b**), 20 μm for (**c**) and 10 μm for (**d**)

We tested whether selectively injured motor neurons upregulate the expression of EphB1, a potential signalling partner for astrocytic ephrin-B1. First, we analysed EphB1 immunoreactivity (IR) in the facial motor nuclei (FMN) of wild type (WT) mice at 1, 7, 14 and 28 days following unilateral facial axotomy in comparison to the unlesioned side (Fig. 1a, b). Neurons in the FMN were identified by NeuN immunostaining^32^, of which 98% represent motor neurons displaying a large soma and distinct large nucleus^33, 34^. Cells showing this nuclear morphology were also ChAT positive, displayed cytoplasmic (Fig. 1b,c) and also surface EphB1 IR as indicated by pre-permeabilisation immunolabelling (Fig. 1d). As early as day 1, there was a 2.57 ± 0.39-fold increase in the number of EphB1/NeuN positive neurons in the ipsilateral (IL) FMN over that of the contralateral (CL) unlesioned side (*p* < 0.01; Fig. 1e, Supplementary Fig. 1). These fold changes in EphB1 IR neurons further increased at day 7 (4.53 ± 0.40-fold, *p* = 0.016; Fig. 1e) and remained significantly raised until day 14 (*p* = 0.045; 2.16 ± 0.14-fold; Fig. 1e) when compared to day 1. In contrast, on the CL side, the number of EphB1-positive neurons remained constant over time (Supplementary Fig. 1). The proportion of EphB1-expressing neurons was also analysed to address the potentially confounding effect of axotomy-induced neuronal loss at later time points. A total of 61.27 ± 4.60% of neurons expressed EphB1 at day 1, and this proportion further increased by day 7 to 93.31 ± 2.48% (*p* < 0.05) before decreasing (day 14: 84.92 ± 3.41%; Supplementary Fig. 1).

### Ephrin-B1 and pSTAT3 levels increase post-axotomy in vivo

We analysed whether astrocytes express ephrin-B1 ligand, a binding partner for EphB1, following facial axotomy-induced motor neuron damage in the FMN, providing a theoretical basis for EphB1 evoked neuron-to-astrocyte signalling. We observed that ephrin-B1 is expressed in glial fibrillary acidic protein (GFAP)-positive reactive astrocyte processes (Fig. 1c,d). We quantified this response by analysing EphB1 and ephrin-B1 protein levels in axotomised FMN tissue homogenates by western blotting (Fig. 1f; Supplementary Fig. 8). Both EphB1 and ephrin-B1 levels were significantly raised to 1.98 ± 0.09-fold (*p* = 0.006) and 1.64 ± 0.06-fold (*p* = 0.0004), respectively, compared to the non-axotomised CL side (Fig. 1g). Western blot (WB) analysis using the same samples also revealed that this coincides with a threefold increased (*p* = 0.034) level of phosphorylated STAT3 (pSTAT3), a key regulator of early astrocyte activation^1, 35^ (Fig. 1f, g). These findings raise the hypothesis that neuronal EphB1 interacts with astrocytic ephrin-B1 to trigger astrocyte reactivity through STAT3 activation.

### EphB1 induces astrocytic STAT3 signalling

**Fig. 2:**
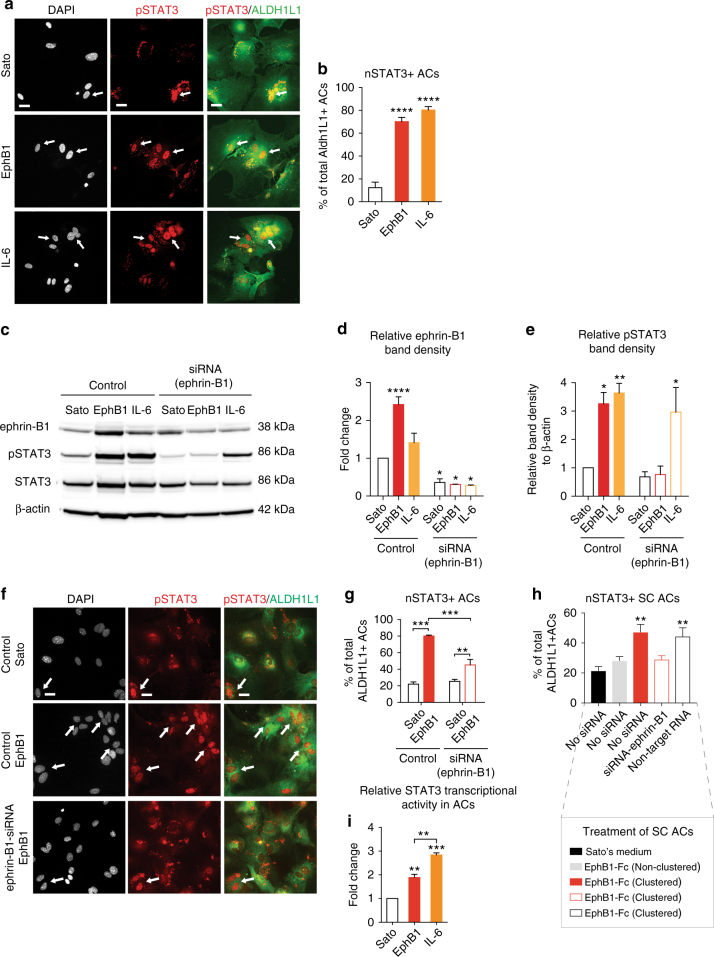
EphB1 acts via ephrin-B1 to induce astrocytic STAT3 activation, and increases transcriptional activity. **a** Immunofluorescence showing nuclear localisation of pSTAT3 (nSTAT3) in ALDH1L1-positive astrocytes (ACs) untreated in Sato’s medium or stimulated with EphB1 or IL-6. **b** Graph shows the percentage of astrocytes displaying nSTAT3 (*n* = 6 cultures from 6 mice; **** *p* ≤ 0.0001, F = 370). **c** WBs of ephrin-B1, total STAT3, pSTAT3 and β-actin, comparing protein levels following no treatment (Sato) and EphB1 or IL-6 treatments of non-transfected control astrocytes with ephrin-B1 siRNA silencing (see also Supplementary Fig. 9). **d**, **e** Graphs show relative band densities of ephrin-B1 (**d**) and pSTAT3 (**e**) to values of non-transfected control astrocytes in Sato’s medium. Data expressed as fold change after normalised to β-actin band density. *N* = 3 cultures from six mice; *p ≤ 0.05; **p ≤ 0.01; ***p = 0.0001, *p = 0.025, *p = 0.015, *p = 0.012, for ephrin-B1, F = 38.2 and *p = 0.013, **p = 0.0044, *p = 0.031 for pSTAT3, F = 9.914; Dunnett’s test. **f** Images showing the extent of nSTAT3 IR in ALDH1L1-positive cortical astrocytes in Sato’s medium and after stimulation with EphB1 with/without ephrin-B1 silencing. **g** Proportion of cortical astrocytes showing nSTAT3 IR 24 h after EphB1 treatment with/without ephrin-B1 silencing (*n* = 4, 5, 5, 4 cultures from 6 mice, respectively; ***p ≤ 0.001. **p ≤ 0.01, F = 63.44). **h** Graph represents the proportion of nSTAT3 labelling in spinal cord (SC) astrocytes following non-clustered or clustered EphB1 treatment alone, with ephrin-B1 siRNA or with non-targeting RNA (*n* = 4, 3, 3, 3 cultures from 6 mice; **p = 0.002, **p = 0.004, respectively, F = 8.78; Dunnett’s comparisons to untreated astrocytes in Sato’s medium). **i** Dual Luciferase assay showing STAT3-driven transcriptional activity in astrocytes following stimulation with EphB1 or IL-6. Graph shows the fold increase of relative luciferase-reporter activity measured by bioluminescence in EphB1-induced or IL-6-induced astrocytes over untreated samples (*n* = 3 cultures from 6 mice; **p ≤ 0.01, ***p ≤ 0.001, F = 124). One-way ANOVA with Bonferroni correction was used if not stated otherwise. Data is expressed as mean ± SEM. Scale bar: 15 μm for (**a**), 20 μm for (**f**)

To examine whether EphB1 induces astrocyte activation through STAT3 phosphorylation and nuclear translocation, we initially used purified mouse cortical astrocyte cultures. Astrocytes were treated with pre-clustered EphB1-Fc in Sato’s serum-free medium to mimic the membrane bound dimerisation of EphB1 receptors on the neuronal surface. First we defined the optimal time point for this treatment in order to avoid a masking effect on STAT3 phosphorylation induced by serum-free conditions. We analysed whole-cell lysates by WBs using antibodies recognising STAT3 phosphorylated at Tyr705 (Supplementary Fig. 2). Increased pSTAT3 band density was apparent after 6 h in serum-free Sato’s media (Supplementary Figs. 2a, c and 10), defining the maximum duration of the EphB1 treatment. Within this time window we have examined the optimal duration of treatment by incubating astrocytes in EphB1 at a recommended dose of 10 μg ml^−1^ for 0.5, 1 or 5 h (R&D Systems). At 5 h, a significant 3.4 ± 0.37-fold increase was observed in pSTAT3 band density levels when compared to non-treated astrocytes, which was comparable to that seen for the canonical STAT3 activator IL-6 (Supplementary Figs. 2b,d and 10). Dose–response analysis within a range of 0.0001 to 10 μg ml^−1^ of EphB1 revealed dose-dependent phosphorylation with a plateau between 5–10 μg ml^−1^ (Supplementary Figs. 2e, g and 10). Guided by these findings in our following experiments we used 10 μg ml^−1^ of EphB1 for 5 h to induce astrocyte responses after 1 h of serum starvation if not stated otherwise (Supplementary Fig. 2a–d). We then assessed whether this also affects the degree of nuclear translocation of STAT3. Cultured astrocytes were immunolabelled for the pan-astrocytic marker aldehyde dehydrogenase 1 family member L1 (ALDH1L1) and astrocytes displaying nuclear IR for pSTAT3 (nSTAT3) were counted (Fig. 2a). EphB1 treatment significantly increased the proportion of ALDH1L1-positive astrocytes containing nSTAT3, which rose from 12.37 ± 1.79% in untreated astrocytes to 70.24 ± 2.49% (*p* ≤ 0.0001) after treatment (Fig. 2b). The positive control, IL-6 induced a comparable increase (80.26 ± 1.25%). Taken together, these data suggest that EphB1 is a potent alternative STAT3 activator.

To explore whether EphB1-induced astrocytic STAT3 activation is specifically mediated via ephrin-B1 signalling, we first transfected astrocytes with ephrin-B1 siRNAs. We found that ~68% lower ephrin-B1 levels assessed by immunoblotting when compared to non-transfected controls (Fig. 2c, d; Supplementary Fig. 9). We then evaluated whether reduction in ephrin-B1 levels prevents EphB1-induced STAT3 activation by assessing pSTAT3, total STAT3 immunoreactive WB bands and nSTAT3 IR by immunocytochemistry. Levels of pSTAT3 measured by quantitative WB from samples of ephrin-B1-silenced astrocytes dropped down to comparable values seen for non-transfected controls (*p* = 0.99; Fig. 2e). Ephrin-B1 silencing also resulted in almost a two-fold decrease in the proportion of EphB1-induced astrocytes displaying nSTAT3 from 80.14 ± 1.06% to 45.23 ± 6.57% (*p* < 0.001; Fig. 2f, g). We then tested the EphB1-induced effect on nSTAT3 IR in spinal cord astrocytes to further explore the relevance of this motor neuron-astrocyte signalling pathway (Supplementary Fig. 3). Like cortical astrocytes, spinal cord astrocytes showed a similar response to clustered EphB1 treatment by a 2.2-fold increase in the proportion of nSTAT3-positive astrocytes (*p* = 0.002), but non-clustered EphB1 had no effect (*p* = 0.539). While pre-treatment with ephrin-B1 siRNA prevented this effect (*p* = 0.437), non-targeting scrambled RNA allowed a significant 2.07-fold increase induced by EphB1 (*p* = 0.004; Fig. 2h, Supplementary Fig. 3a). Taken together, these results confirm that EphB1-induced STAT3 activation in astrocytes is mediated, at least in part, through ephrin-B1-induced reverse signalling. Next we sought to demonstrate whether EphB1 treatment results in STAT3-mediated transcription by performing a dual luciferase assay. We found that EphB1 treatment markedly enhanced STAT3 reporter activity when compared with vehicle-treated astrocytes, showing a 1.89 ± 0.13-fold increase in bioluminescence (*p* < 0.01; Fig. 2i). The positive control, IL-6 also induced a significant response (*p* < 0.001). These findings suggest that EphB1 is a potent alternative activator of astrocytes via STAT3-mediated transcription.

### EphB1 and IL-6 both induce a reactive astrocyte phenotype

We assessed whether EphB1-induced signalling triggers reactive cytoskeletal changes commonly observed following astrocytic STAT3 activation^1, 36^. We visualised the distribution of F-actin by phalloidin labelling and quantified cortical F-actin ring formation, an in vitro phenomenon characterising activated astrocytes^37, 38^. During serum starvation, most astrocytes displayed a flat morphology with parallel actin fibres (Supplementary Fig. 3b). In contrast, the majority of astrocytes stimulated with EphB1 or IL-6 (76.04 ± 2.18% and 72.30 ± 5.48%, respectively) showed thick cortical actin bands, and radial actin filaments extending towards the cell periphery, indicative of a reactive morphology (Supplementary Fig. 3c).

To confirm changes in cytoskeletal protein levels associated with EphB1 or IL-6 induced during astrocyte activation, we assessed GFAP levels^39^ by quantifying WBs (Supplementary Fig. 3d). Both EphB1-induced and IL-6-induced GFAP levels by a 2.38 ± 0.35 and 2.29 ± 0.21-fold level when compared to controls, respectively (Supplementary Fig. 3e). Moreover, we confirmed that EphB1-mediated upregulation of GFAP is STAT3-dependent by using protein lysates of astrocytes cultured from *Gfap-Stat3*-conditional knock-out (CKO) mice. Specifically, CKO astrocytes showed negligible levels of total STAT3, no pSTAT3 IR and GFAP protein levels failed to rise on EphB1 or IL-6 stimulation (Supplementary Fig. 3d,e). Taken together, these results suggest that in vitro EphB1 induces a morphological astrocyte transformation accompanied by increased GFAP expression in a STAT3-dependent manner, resembling IL-6 triggered reactive phenotypic changes.

### EphB1 invokes a STAT3 target-linked astrocyte transcriptome

**Fig. 3:**
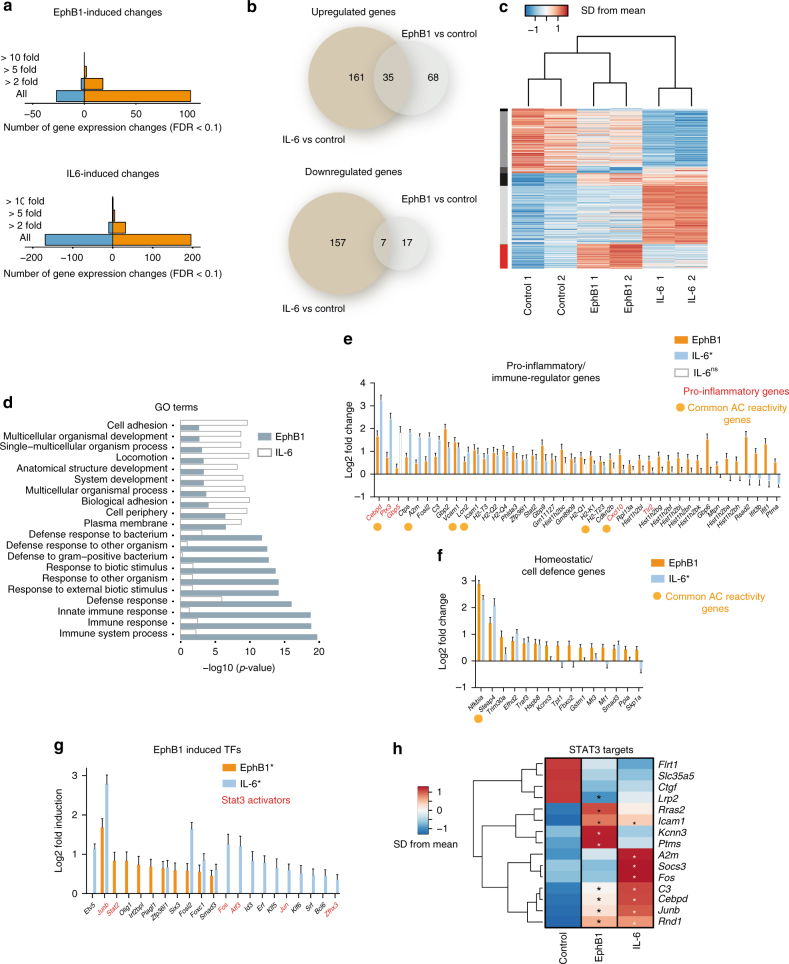
Transcriptome-wide analyses of purified mouse astrocytes treated with EphB1 or IL-6. **a** Graphs show the total of changed transcripts induced by EphB1 or IL-6 treatment. **b** Diagram demonstrating the number of treatment specific and commonly upregulated and downregulated genes (*n* = 2 independent cultures from 6 mice; FDR ≤ 0.1). **c** Unsupervised hierarchical clustering of control astrocytes (ACs), IL-6 and EphB1-treated gene expression profiles demonstrates separation of each experimental group. Heatmaps show the representation of top genes by contribution to Principal Component. Vertical colour bar indicates groupings of genes according to patterns of change in response to EphB1 and IL-6. Gene expression data represent SD from mean of variance stabilised values across rows. Upregulated genes are shown in red, downregulated genes are shown in blue. **d** GO term analysis of significantly induced genes (FDR ≤ 0.1) when comparing EphB1 to IL-6 treatment using the DAVID interface. **e**,**f** Significantly induced transcripts by EphB1 in comparison with IL-6-induced response, representing pro-inflammatory/immune-regulator genes (**e**) or homeostatic/cell defence (**f**) astrocyte reactivity profiles. For IL-6 non-significant changes are represented by empty bars. Orange dots label commonly upregulated transcripts of reactive astrocytes defined by published data^8^. **g** Significant EphB1-induced or IL-6-induced TFs in which STAT3 activators are labelled in red. **h** Heatmaps of STAT3 targets induced by either EPhB1 or IL-6 treatment of astrocytes. Stars represent significantly induced transcripts (*p* ≤ 0.01) and data represent SD from mean. For graphs data shows log2-fold changes in gene expression ± SEM

We then assessed whether EphB1 to ephrin-B1 signalling resulted in a characteristic transcriptomic signature in stimulated astrocytes, reflecting potentially beneficial or harmful aspects of astrocyte reactivity. To do this, we examined EphB1-induced STAT3-dependent or independent changes and compared these to IL-6 (a canonical STAT3 activator) mediated pathways. We explored changes in the astrocyte transcriptome using RNA-seq at 24 h post-stimulation with either EphB1 or IL-6 (2 independent cultures per condition of highly purified (>99%) astrocytes collected from 6 mice). We analysed differentially expressed genes between the responses induced by EphB1 and IL-6 (FDR < 0.1; Fig. 3a). When compared to IL-6, EphB1-induced 68 and 17 non-overlapping in addition to 35 and 7 overlapping upregulated and downregulated transcripts, respectively (Fig. 3b, c). Gene ontology (GO) analysis indicated that the most significant EphB1 specific enrichment was related to the upregulation of intercellular signalling, stress and immune response pathways (Fig. 3d). Meanwhile the largest proportions of IL-6-specific changes represented cell adhesion and locomotion (Fig. 3d). Analysis confirmed major differences between EphB1- and IL-6-mediated gene expression profiles regulating astrocyte reactivity/proinflammatory cascades and homeostatic pathways (Fig. 3e, f; Supplementary Fig. 4). These represented 57 genes among which only 7 transcripts belonged to common astrocyte reactivity genes^8, 40, 41^. Among the 42 significantly upregulated EphB1-induced transcripts with immune/inflammatory profile 30 genes were differentially changed from those seen for IL-6. These included proinflammatory regulators *Cebpd* and *Ptx3*, which were induced to lesser extent by EphB1 when compared to IL-6-induced changes (Fig. 3e). Importantly, EphB1 more significantly induced transcripts with immune-modulatory roles, such as *Ifit3b*, *Ifit1*, *Ptma* and *Trim30a*, *Nfkbia* with additional cell defence profiles than IL-6 (Fig. 3e, f). In addition, 10 out of 15 significantly upregulated genes with homeostatic roles were induced to higher levels by EphB1 than by IL-6, including *Kcnn3*, *Tpt1*, *Gstm1*, *Mt3* and *Mt1* (Fig. 3f).

**Fig. 4:**
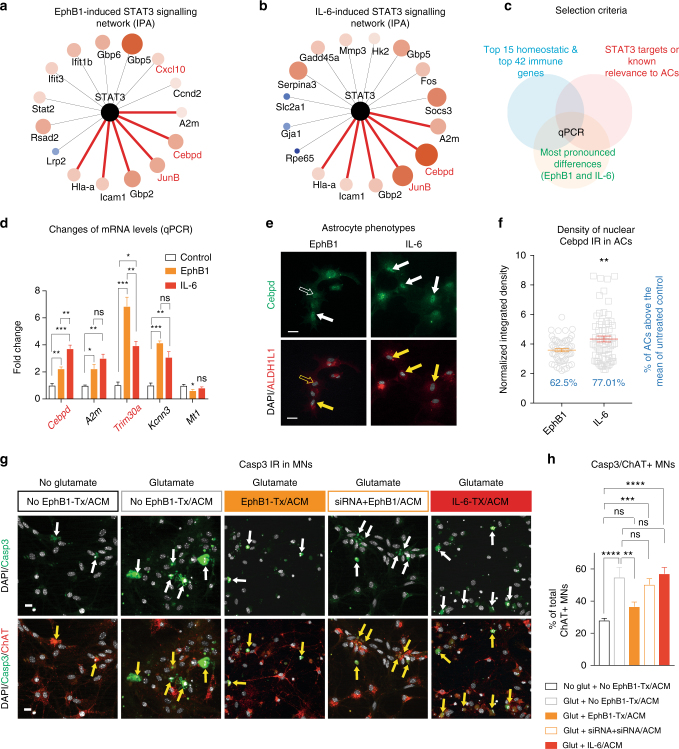
EphB1 induces a neuroprotective transcriptional programme in astrocytes which is distinct to IL-6 treatment. **a**,**b** Predictive EphB1/IL-6 signalling to the STAT3 transcriptomic network. Connections are based on the provided evidence by Ingenuity Pathway Analysis. Red lines indicate connections of STAT3 to inflammatory transcripts, which are overlapping between EphB1-induced and IL-6-induced networks. Both larger node size and darker orange colour indicate increased transcript induction while smaller node size and darker blue colour codes decreased gene expression. **c** Diagram indicates selection criteria for qPCR based validations. **d** Graph shows mean fold changes of transcripts in astrocytes (ACs) following EphB1 or IL-6 treatments normalised to untreated controls (*n* = 3 cultures from 6 mice; **p = 0.003, F = 58.44 for Cebpd and **p = 0.009, F = 44.1 for Trim30a between EphB1 and IL-6 treated groups; one-way ANOVA with Bonferroni post hoc test). **e** Fluorescence images demonstrate Cebpd IR in EphB1-induced or IL-6-induced ALDH1L1-positive astrocytes. **f** Dot plot graph shows relative integrated density measurements of nuclear Cebpd IR to the mean of control values after normalisation to background densities (*n* = 55 for EpHB1, *n* = 67 for IL-6 from three astrocyte cultures of six mice; **p = 0.002, unpaired *t*-test). Percentages represent the proportion of astrocytes with higher values than the control threshold. **g** Immunofluorescence images of motor neurons (MNs) displaying cleaved-caspase3 IR in glutamate toxicity assays, in which the effect of AC conditioned media (ACM) was examined. ACM derived from astrocytes receiving various treatments: untreated or treated astrocytes by EphB1 with or without pre-incubation by ephrin-B1 siRNA and also by IL-6. **h** Graph demonstrates the mean proportion of glutamate (100 μM) toxicity induced cleaved-caspase3 positive spinal cord motor neurons and the influences imposed on this by various ACM (*n* = 6, 5, 4, 4, 5 motor neuron populations from six mice in two independent experiments; ****p ≤ 0.0001, ^ns^ *p* = 0.668, ***p = 0.0007 in comparison with non-glutamate treated negative controls or **p = 0.019, ^ns^ *p* = 0.832 and ^ns^ *p* = 0.997, F = 16.41 in comparison with glutamate and ACM treated control, one-way ANOVA with Tukey’s post-test). Scale bar: 20 μm

Next, we assessed whether EphB1 triggered inflammatory signalling specifically enhances gene expression within the STAT3 transcriptional network that has been described to mediate protective pathways^2, 3, 36^. Among the EphB1-induced transcription factors we identified STAT3 inducers, *Stat2* and *JunB*, which were differentially expressed when compared to the IL-6-mediated response (Fig. 3g). With regard to putative and known STAT3 targets, the highest EphB1-induced expression changes included homeostatic genes (*Kcnn3, Ptms*) or immune-modulatory transcripts (*Icam*) (Fig. 3h). A specific analysis of the mouse STAT3 regulatory network identified by Ingenuity Pathway Analysis (IPA) revealed six common transcripts induced by both EphB1 and IL-6. Among these major inflammation associated genes *Cebpd*, *A2m* and *JunB* were induced by EphB1 to a lesser degree (Fig. 4a, b). In summary, although both EphB1 and IL-6 activate STAT3 pathways, the transcriptional profile suggests that EphB1 shifts STAT3-mediated processes from a deleterious proinflammatory spectrum towards a more beneficial inflammatory transcriptional profile.

We validated key differences found in the transcriptomic networks which discriminate EphB1-induced from IL-6-induced phenotypes using qPCR. For this we used samples from three independent cultures of astrocytes with technical duplicates deriving from six mice each. We selected 5 transcripts that represent the most pronounced differences between EphB1-induced and IL-6-induced profiles among the 15 homeostatic and 42 inflammatory genes, which have either published relevance to astrocytes or are putatively regulated by STAT3 (Fig. 4c). The most significant findings were a lower expression of a major proinflammatory regulator, *Cebpd* and also a nearly two-fold higher expression of *Trim30a*, a negative inflammatory element in EphB1-induced astrocytes (Fig. 4d). These findings were further supported by our immunocytochemical analysis, showing decreased Cebpd activation by a 20.8% reduction in integrated density (IntDen) of nuclear Cebpd IR in astrocytes when compared to that observed from IL-6-induced astrocytes (*p* = 0.002; Fig. 4e, f). Unlike *Trim30a* mRNA expression levels, Trim30 protein levels were not significantly changed, at least, 5 h post-stimulation by EphB1 (Supplementary Figs. 5 and 10).

Having confirmed that EphB1 signalling specifically induces a beneficial astrocyte phenotype defined by transcriptional and immuncytochemical analysis, we then sought to establish if these changes were functionally consequential. To do so, we evaluated cleaved-caspase3/ChAT-positive spinal cord motor neurons treated by astrocyte-conditioned media (ACM) in excitotoxicity assays (Fig. 4g). Glutamate induced an increase in the proportion of cleaved-caspase3-labelled motor neurons (58.8 ± 5.75%) in the presence of ACM. This excitotoxic insult was partially rescued by ACM collected from EphB1-treated astrocytes (35 ± 2.91; *p* = 0.019; Fig. 4h), but not by ACM obtained from treated astrocytes that were pre-transfected with ephrin-B1 siRNA (53.28 ± 5.56%; *p* = 0.832; Fig. 4h). ACM from IL-6 treated astrocytes had no preventative effect (*p* = 0.997). These results support our transcriptomic data indicating a neuroprotective phenotype of EphB1-treated astrocytes.

### Neuronal EphB1 and astrocyte STAT3 responses fail in ALS mice

**Fig. 5:**
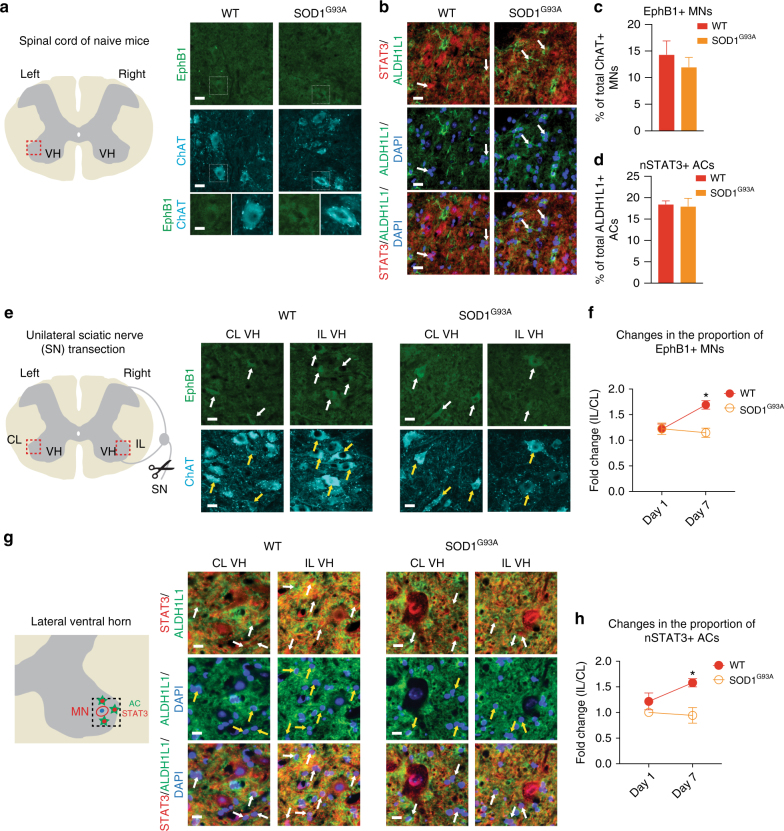
EphB1 and STAT3 expression patterns in the ventral horn of wild type and SOD1^G93A^-ALS mice. **a** Schematic diagram and immunofluorescence images showing very low levels of EphB1 immunolabelling in ChAT-positive motor neurons (MNs) in the ventral horn (VH) of unlesioned wild type (WT) and SOD1-mutant mice. White boxes represent the corresponding cells magnified in the insets for each group. **b** Immunofluoresence demonstrating ALDH1L1-positive astrocytes (ACs) with nuclear STAT3 (nSTAT3) immunostaining (arrows) in unlesioned WT and SOD1-mutant mice. **c** Graph demonstrates the mean of the EphB1-labelled proportion of ChAT-positive motor neurons in the two groups (*n* = 4; *p* = 0.476, unpaired *t*-test). **d** Graph demonstrates the mean of the nSTAT3-labelled proportion of ALDH1L1-positive astrocytes in the two groups (*n* = 4; *p* = 0.821, unpaired *t*-test). **e** Schematic diagram and immunofluorescence images demonstrating EphB1 positivity (arrows) corresponding with ChAT-labelled motor neurons in the ipsilateral (IL) and contralateral (CL) VH of mice following right-sided sciatic nerve (SN) transection. **f** Graph shows the mean values of fold changes in the proportion of EphB1-labelled motor neurons in the IL VHs when normalised to the CL side (*n* = 4, *p* = 0.956 for day 1; *n* = 3, *p = 0.011 for day 7, unpaired *t*-test). **g** Schematic diagram and immunostained tissue sections demonstrating ALDH1L1-positive astrocytes with nSTAT3 IR (arrows). **h** Graph shows mean values of fold changes in the proportion of nSTAT3-positive astrocytes in the IL VHs normalised to the CL side (*n* = 3 controls, *n* = 4 SOD1, *p* = 0.227 for day 1; *n* = 4, *p = 0.010 for day 7, unpaired *t*-test). Data is expressed as mean ± SEM. Scale bar: 40 μm (20 μm for insets)

Having established that EphB1 and STAT3 are important in motor neuron-to-astrocyte communication, resulting in a protective reactive astrocyte phenotype, we then hypothesised that such signalling is perturbed in ALS. Initially, we examined whether motor neurons could potentially trigger this beneficial astrocyte response in a mouse ALS model. Using tissue sections from lumbar spinal cords of symptomatic SOD1^G93A^-ALS mice and age-matched WT controls, we examined EphB1 IR in motor neurons identified by ChAT labelling in the latero-ventral horn innervating the lower limbs (Fig. 5a). Within the 100 μm of motor neuron nuclei, we also analysed nSTAT3-/Aldh1L1-positive astrocytes (Fig. 5b). EphB1 IR was negligible in naive mice in general. The proportion of EphB1 immunoreactive motor neurons and nSTAT3-positive astrocytes did not increase in the symptomatic SOD1^G93A^-ALS mice and was comparable to that seen in WT controls (Fig. 5c, d). We then evaluated whether motor neurons and astrocytes would respond to additional motor neuron injury in SOD1^G93A^-ALS mice induced by unilateral sciatic nerve transection at days 1 and 7 when compared to the unlesioned CL side (Fig. 5e–h). At day 1, the responses were subtle and comparable between the groups (*p* = 0.227, unpaired *t*-test; Fig. 5f, h). However, at day 7 in SOD1^G93A^-ALS mice there was no further increase in the number of EphB1-positive motor neurons and nSTAT3-labelled astrocytes, unlike in their WT counterparts with a 1.69-fold and 1.58-fold increase of EphB1-positive motor neurons and nSTAT3 immunoreactive astrocytes post-axotomy (*p* = 0.011 and *p* = 0.010, respectively; Fig. 5f, h). Collectively, our data suggest a failure of astrocytic STAT3 activation in response to neuronal injury in our ALS mouse model. This raises the question of whether an intrinsic dysfunction in astrocytes underlies this impaired astrocyte activation.

### Pertubations in ALS patient-derived astrocyte reactivity

**Fig. 6:**
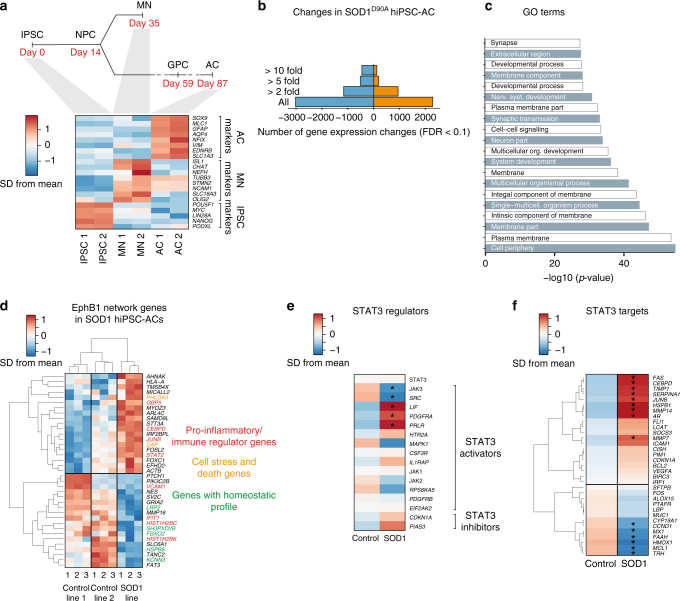
Transcriptome-wide analysis in enriched human SOD1^D90A^ ALS patient-derived iPSC-astrocytes. **a** Heatmaps indicating exclusive enrichment of astrocyte (AC)-specific genes vs. hiPSC-specific and motor neuron-specific transcripts. **b** The number of gene expression changes (FDR ≤ 0.1) vs. controls (*n* = 3 independently converted astrocyte cultures from hiPSCs; see Supplementary Table 1 for information on patient lines). **c** GO term analysis of genes significantly upregulated when compared to controls using the DAVID interface. **d** Most significantly downregulated or induced transcripts in control or SOD1 hiPSC-astrocytes, which are overlapping with EphB1-induced mouse astrocyte genes. Known common pro-inflammatory/immune-regulator, cell stress/death genes and transcripts with homeostatic profile are labelled in red, orange and green, respectively. **e**,**f** Heatmaps demonstrating upregulation or downregulation of STAT3 inducers and inhibitors (**e**) and the STAT3 target profile (**f**) in control and SOD1-mutant hiPSC-astrocytes. Stars in **e**,**f** indicate significantly changed genes (FDR ≤ 0.1). For heatmaps gene expression data represent SD from mean of variance stabilised values across rows

We then examined potential alterations in astrocyte reactivity in human ALS, focusing on the EphB1 and STAT3-regulated transcriptome. We employed highly enriched (99%; Supplementary Fig. 6a, b) cultures of patient-specific SOD1^D90A^ hiPSC-astrocytes generated by our previously published methods^42^. For RNA-seq experiments we used three independently differentiated hiPSCs-astrocyte cultures from two healthy controls and one SOD1^D90A^ ALS patients. For mRNA and functional validations of the gene expression profiles, we then used 3–13 independently differentiated hiPSCs-astrocyte cultures from three healthy controls and two SOD1^D90A^ ALS patients. Importantly, this included an isogenic pair (Supplementary Table 1). Transcriptome-wide profiling indicated exclusive enrichment of astrocyte-specific genes vs. iPSC and motor neuron markers (Fig. 6a). Initial analysis revealed 1909 upregulated and 2513 downregulated transcripts in the SOD1 lines vs. controls (Fig. 6b). GO terms and individual gene analysis revealed that there was concordance in the main upregulated proinflammatory transcripts between SOD1-mutant hiPSC-astrocytes and the astrocyte-specific translational profile in SOD1-mutant ALS mice^25^ (Fig. 6c). Interestingly, 18% of reactive astrocyte genes identified in mouse models^8^ were also similarly changed in SOD1-mutant hiPSC-astrocytes and 8% of genes found altered in microdissected astrocytes from SOD1 mice^43^ were also changed in hiPSC-astrocytes. This reflects similarities existing between human and mouse astrocyte responses despite differences in species and experimental platforms. Specifically, a proportion of altered genes in SOD1 astrocytes were identified as EphB1 response genes in our mouse system (Fig. 6d). Among these, there were five major significantly upregulated transcripts with detrimental or proinflammatory effect, which were more representative of IL-6-induced genes than of those activated by EphB1 in mouse astrocytes (*PHLDA3, CEBPD, JUNB, STAT2, GBP5*). Importantly, 5 EphB1-signalling associated transcripts with protective or homeostatic effects had decreased expression in SOD1-mutant astrocytes, including *HSPB8*, *KCNN3*, *LRP2*, *FBXO2*, *SH3PXD2B* (Fig. 6d).

To investigate whether downregulated transcripts associated with the EphB1 pathway also affect STAT3 signalling in SOD1-mutant hiPSC-astrocytes, we compared transcriptomic changes with the human STAT3 interactome. This showed that among the five significantly changed STAT3 activators *JAK3* and *SRC* were downregulated, meanwhile *LIF*, *PDGFRA*, *PRLR* were upregulated (Fig. 6e). We also identified nine significantly induced STAT3 targets while six were decreased (Fig. 6f). Altogether, the RNA-seq data raised the hypothesis of a specific dysregulation of EphB1-induced reactivity genes (Fig. 6d–f).

**Fig. 7:**
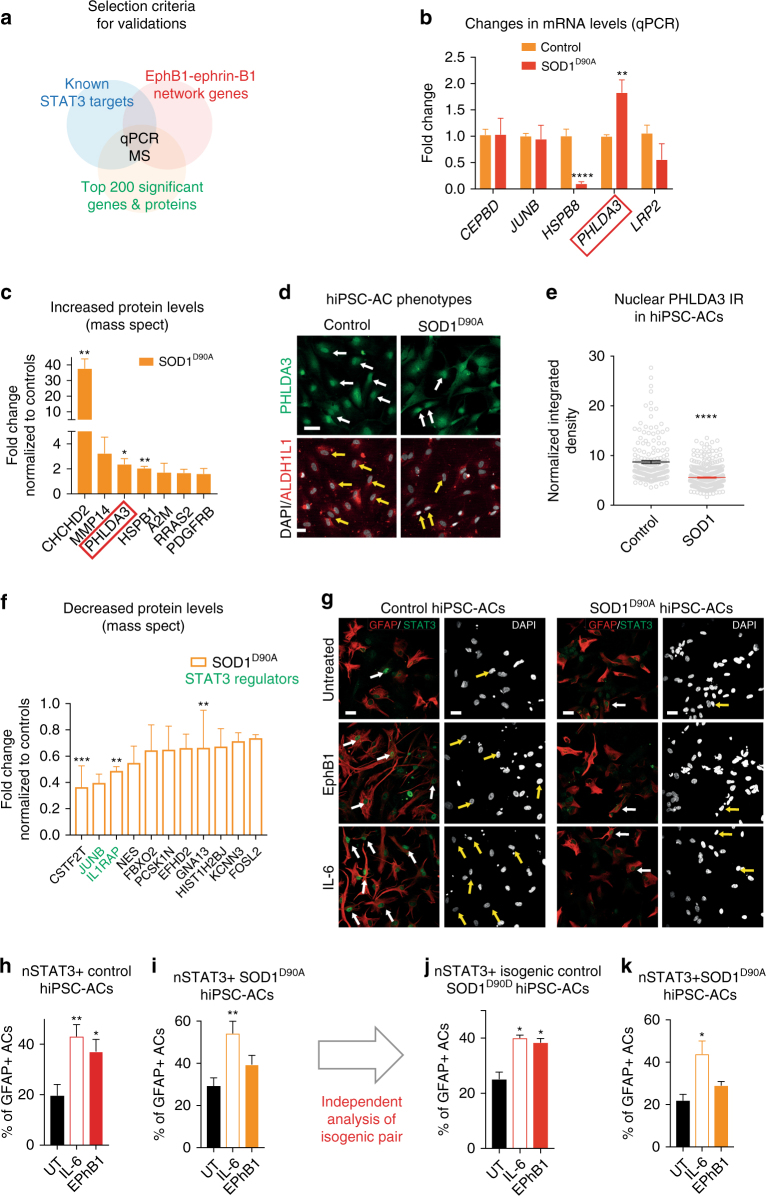
Validation of dysregulated transcripts within the EphB1–STAT3 network and the failure of STAT3 activation in human SOD1^D90A^ iPSC-astrocytes. **a** Diagram of selection criteria for validation of gene expression data. **b** Graph indicates mean fold changes of mRNA of top transcripts when normalised to controls (*n* = 8 control and 7 SOD1-mutant independently converted hiPSC-astrocyte (AC) cultures, Supplementary Table 1; ****p ≤ 0.0001, **p = 0.003; unpaired *t*-test). **c** Graphs show mean fold changes of increased protein levels normalised to the mean of controls when measured by mass spectrometry (MS) (*n* = 3 independent cultures of SOD1-mutant hiPSC-astrocytes vs. control astrocytes; **p = 0.003, *p = 0.039, **p = 0.0015, unpaired *t*-test). **d** PHLDA3 IR pattern in ALDH1L1/DAPI-labelled control and SOD1-mutant hiPSC-astrocytes. **e** Dot plot graph shows integrated density measurements of nuclear PHLDA3 IR in astrocytes after normalised to background. *N* = 378 cells from three SOD1 patient-derived hiPSC-astrocytes and 181 cells from two control patients; ****p ≤ 0.0001, unpaired *t*-test. **f** Graphs shows mean fold changes of decreased protein levels normalised to the mean of controls when measured by MS (*n* = 3 independent cultures of SOD1-mutant hiPSC-astrocytes vs. control astrocytes, ***p = 0.0001, **p = 0.009, **p = 0.003, unpaired *t*-test). **g** Panels demonstrate immunofluorescence images of GFAP-positive astrocytes with nSTAT3 IR in control and in SOD1 hiPSC-astrocytes. Adjacent panels with DAPI staining illustrate nSTAT3 co-localisation (arrows). **h**–**k** Graphs represent the proportion of nSTAT3-positive cells among the total of GFAP-positive astrocytes in control (**h**) or SOD1-mutant hiPSC-cultures (**i**), which is also independently analysed for the isogenic corrected control/SOD1-mutant pair of hiPSC-astrocyte cultures (**j**, **k**). For **h** and **i**, *n* = 13 and 9 independently converted hiPSC-astrocyte cultures; **p = 0.002, *p = 0.029 for controls, **p = 0.003 for SOD1 hiPSC-astrocytes, one-way ANOVA with Bonferroni test. For **j** and **k**, *n* = 2 independently converted astrocyte cultures for the isogenic control and *n* = 3 for the SOD1-mutant pair; *p = 0.027 for IL-6 and *p = 0.038 for EphB1 in controls; *p = 0.025 for IL-6 and *p* = 0.777 for EphB1 in SOD1 astrocytes; F = 21.95 and F = 7.8, respectively; one-way ANOVA with Bonferroni test. Data expressed as ± SEM. Scale bar: 30 μm

We validated five significantly altered transcripts belonging to EphB1 and/or STAT3 networks by qPCR using eight and seven independently differentiated hiPSCs-astrocyte cultures from three healthy controls and two SOD1^D90A^ ALS patients, respectively (Fig. 7a; Supplementary Table 1 and 2). A 1.83-fold induction of *PHLDA3* (*p* = 0.003), a gene recently reported to be linked to degenerative mechanisms, and a 10-fold downregulation of *HSPB8* with a protective profile (*p* < 0.0001; Fig. 7b) were found.

We next performed Tandem Mass Tag (TMT)-mass spectrometry (MS) on whole-cell lysates of six independently differentiated hiPSCs-astrocyte cultures from three healthy controls and two SOD1^D90A^ ALS patients, including an isogenic pair (Supplementary Table 1; Supplementary Data File). By focusing on the most differentially expressed proteins (Fig. 7c, f), we confirmed that PHLDA3 exhibited increased expression in SOD1-mutant hiPSC-astrocytes (2.40-fold over controls, *p* = 0.039), corresponding with its higher transcriptional expression analysed by RNA-seq and qPCR (Fig. 7c; Supplementary Fig. 7). In addition to higher PHLDA3 protein levels, the reduction of IntDen measurements of PHLDA3 nuclear IR in SOD1-mutant hiPSC-astrocytes indicated a shift in intracellular distribution, suggesting activation of degeneration pathways^44, 45^ (Fig. 7d, e). Although the level of a mitochondrial protein CHCHD2 was significantly high, over 30-fold in SOD1-mutant astrocytes, it did not correlate with increased transcription, and may indicate possible protein accumulation. The proteomic analysis focussed on the STAT3 network members demonstrated a significantly lower, 0.49-fold level of the STAT3 activator IL1RAP (*p* = 0.009; Fig. 7f, Supplementary Fig. 7). These data raise the hypothesis that EphB1-induced STAT3 activation and/or its downstream pathways are dysregulated. We tested this hypothesis by assessing nuclear translocation of STAT3 IR in ALS patient-specific GFAP expressing hiPSC-derived SOD1^D90A^-astrocytes compared to healthy control astrocytes in the presence or absence of EphB1, again using IL-6 as a positive control. We used 9–13 independently differentiated hiPSCs-astrocyte cultures from three healthy controls and two SOD1^D90A^ ALS patients, including an isogenic pair. We first identified the most effective concentration of EphB1 in human astrocyte cultures (Supplementary Fig. 6). We then demonstrated that EphB1-induced nSTAT3 translocation is impaired in SOD1 hiPSC-astrocytes when compared to IL-6-triggered response (*p* = 0.003; Fig. 7g–i). Indeed the proportion of nSTAT3 IR in SOD1-mutant astrocytes did not differ from those in untreated control cultures (*p* = 0.398; Fig. 7i). Conversely, significant 1.88-fold and 2.18-fold responses were seen in healthy controls for EphB1 and IL-6, respectively, (*p* = 0.002 and *p* = 0.029; Fig. 7h). Importantly, we also separately validated these findings in SOD1-mutant hiPSC-astrocytes and isogenic (genetically corrected) control hiPSC-astrocytes using qPCR, MS and STAT3 activation assays (Fig. 7j, k, Supplementary Fig. 7). These data cumulatively suggest an intrinsic failure of the EphB1–ephrin-B1–STAT3 signal transduction pathway in SOD1-mutant iPSC-astrocytes. In summary, we have demonstrated that astrocyte cell autonomous mechanisms contribute to diminished EphB1–ephrin-B1-mediated reverse signalling and STAT3 activation in ALS astrocytes.

## Discussion

It is a long-held view that astrocyte activation in injury involves inflammatory responses and cytokine signalling, through a cascade induced by microglial reactivity and immune cells^4, 11, 46^. However, early injury cues that define an astrocyte phenotype favourable for repair processes remain elusive. We provide both in vivo and direct in vitro evidence that an early complementary route exists for astrocyte activation by neuronal injury cues through EphB1-induced signalling. Our in vitro paradigm confirms that EphB1 that is expressed on motor neurons directly triggers astrocyte transformation both at morphological and transcriptional levels, resembling a reactive astrocyte phenotype. Similar neuron-astrocyte signalling may also exist in other CNS regions, such as the hippocampus, where upregulation of EphB1 expression in axotomised neurons is concomitant with the increase in GFAP IR^17^. There may be several routes by which motor neuron EphB-receptors could potentially induce reverse signalling in astrocytes due to their promiscuous binding to various ligands in different cell types^47, 48^. We confirmed that EphB1 activates astrocytes by inducing ephrin-B1 dependent STAT3 phosphorylation. This is supported by studies in non-neural models, describing the direct recruitment of STAT3 to the intracellular domain of ephrin-B1, resulting in phosphorylation of STAT3 at *Tyr 705* ^49, 50^. It is noteworthy that there are other alternative pathways for reverse signalling through ephrin-B1^20^, potentially leading to a possible crosstalk with the STAT3 signalling network.

Here, we demonstrate that EphB1 not only induces STAT3 phosphorylation in astrocytes but also its nuclear transfer and transcriptional activity concomitant with reactive morphological astrocyte transformation. Transcriptome-wide analyses confirmed and extended these results by demonstrating a unique reactivity profile discriminating the EphB1 response from canonical astrocyte activators, such as IL-6. Notably, this also appears to differ when compared to microarray studies on the profile of lipopolysaccharide and ischaemia-induced astrocyte activation^8, 51^. Our finding prompts speculation on the specific role of EphB1–STAT3-induced astrocyte reactivity and its functional consequences.

STAT3-regulated pathways are emerging as central players in astrocyte-mediated neuronal survival, axon regeneration and synapse recovery in injury-associated inflammatory environments^1–3, 35^. Its role in activating genes that suppress deleterious proinflammatory processes has been also described^52^. In neuronal injuries IL-6 can activate astrocytic STAT3^53^ and may exert a regenerative and anti-inflammatory effect through its membrane bound gp130-linked receptor, IL-6R. However, neuronal insults are also known to induce a significant deleterious proinflammatory response via the IL-6 soluble receptor, sIL-6R^31^ and also by various cytokines such as tumour necrosis factor-alpha (TNF-α) and interleukin(IL)-1β^4^. Thus cellular and molecular determinants that orchestrate the balance between the toxic and neuroprotective effects have remained unclear. We propose that early neuronal EphB1 expression, in particular through STAT3, has the potential to promote protective, anti-inflammatory or immune-modulatory pathways via ephrin-B1 reverse signalling. Although ephrin-B reverse signalling has previously been reported to induce peripheral immune system activation^19^, our data uncover its association with an inflammation-modulatory transcriptional response, suggesting another role for the Eph–ephrin interactions in the central nervous system. Beyond some shared classical reactivity genes^8, 54^, EphB1-induced unique transcriptional changes linked to regenerative profiles. In contrast to IL-6, EphB1 favoured activation of transcripts and networks suggesting a shift from proinflammatory activation towards an immune-modulatory or anti-inflammatory arm of the STAT3 pathway. For IL-6 these differences may be explained by the sIL-6-R-mediated proinflammatory effect despite protective STAT3 signalling activated through the membrane bound IL-6R. The induction of other pathways might have also modulated the astrocyte transcriptome profiles, leading to a more favourable overall outcome for EphB1. Overall, this EphB1 activated phenotype is characterised by diminished *Cebpd* and increased *Trim30a* expression, and induced a significantly less cleaved-caspase3 IR in motor neurons when compared to the IL-6-induced astrocyte phenotype, suggesting a protective role. Given recent discoveries of the favourable impact of astrocytic STAT3 signalling on neuronal function, this novel astrocyte activation route is of relevance not only for neuroprotective effects but also for supporting synapse and axon repair^55^. We speculate that EphB1-mediated responses serve to counterbalance the neuronal injury response, including IL-6R or Megf10 signalling, which themselves trigger initial synapse elimination and debris clearance^56, 57^. In support of this notion, the time course of the increased EphB1 IR in damaged motor neurons corresponds with the restorative phase following motor neuron axotomy in our paradigm^58^.

Our work suggests a rapid signal transduction via EphB1 from neurons to astrocytic STAT3 activation, which appears to be a hallmark of the protective astrocyte phenotype^1, 3, 36^. Despite increasingly recognised astrocyte heterogeneity^59^, in our paradigm the conserved response of astrocytes throughout their cortical, brainstem and spinal cord origin indicates a unified mechanism. This non-region specific response was also suggested by the protective effect on spinal motor neurons by supernatants of EphB1-induced and STAT3-activated cortical astrocytes. This is also supported by observed STAT3 expression in various regenerating CNS regions following neuronal insults^1, 3, 60^.

The elucidation of this default astrocyte response helped to address whether astrocytic reactivity and its potential effects in counterbalancing neuroinflammatory or neurotoxic events fail in neurodegenerative disease. This is an increasingly relevant issue in ALS, in which raised IL-6 levels^61^ and widespread inflammatory activation has been thought to fuel non-cell autonomous pathology, being ultimately detrimental to motor neurons^26^. So far attempts have been limited to explore these issues due to the lack of human models suitable to dissect astrocyte-specific changes. We show for the first time that human SOD1 ALS patient-derived iPSC-astrocytes have an altered transcriptome specifically related to the machinery of astrocyte reactivity. This is supported by a previous study in SOD1-mutant ALS mice, demonstrating diminished GFAP expression in astrocytes induced by motor neuron axotomy^28^. Our findings demonstrate the power of integrated interspecies experimental models to generate high confidence findings of altered astrocyte states. In particular, we confirmed a partial intrinsic dysregulation of the EphB1-mediated pathway and the loss of the protective astrocyte phenotype relevant to one of the predominantly affected anatomical regions in ALS, by a transcriptomic approach combined with MS and/or immunolabelling across in vivo and in vitro model systems.

In correlation with transcriptional changes, increased levels of PHLDA3 and the impairment in STAT3 activation in ALS hiPSC-astrocytes are important findings to further explore in future studies. PHLDA3 is a recently discovered member of p53-dependent signalling and is activated in endoplasmic reticulum stress^44, 62^. However, whether this indicates a novel example of an astrocyte-specific degeneration process in ALS has yet to be confirmed. The failure of STAT3 activation in astrocytes is a compelling finding. Although, unlike in mouse models, proinflammatory regulators such as CEBPD did not feature primarily in cultured human ALS iPSC-astrocytes, it is striking that EphB1–STAT3-mediated protective inflammatory or anti-inflammatory pathways are inadequately activated both in our mouse and human ALS models. This may also allow an overall proinflammatory profile with motor neuron vulnerability well recognised in SOD1 ALS mice^25, 63^ and human ALS, and could provide some answers as to why global suppression of inflammation has failed in human clinical trials. Indeed, using a combination of mouse models and human iPSC models, such as demonstrated in our paradigm, is required to confidently identify relevant astrocytic targets for inflammatory modulation as a potential treatment strategy for neurodegenerative diseases^64, 65^. Therapeutic options may include combined pathway-specific approaches that augment the protective or anti-inflammatory STAT3 downstream signalling in astrocytes^1, 3, 36, 66^ and inhibit detrimental IL-1β or TNF-α induced NF-kB-dependent proinflammatory activation^15, 63, 67, 68^.

In conclusion, we identified EphB1 as a novel and important trigger of early astrocyte response pathway partially through activation of STAT3, which mediates restorative processes. We provide the first evidence that in human ALS, astrocytes cell-autonomously have impaired EphB1-mediated STAT3 activation. This constitutes a novel example of astrocyte ‘loss of function’ in ALS^5, 6^. Our study adds to the understanding of astrocyte responses in injury and neurodegeneration, and potentially highlights new neuroprotective therapeutic targets.

## Methods

### Surgical facial nerve axotomy

All experimental procedures were carried out under the UK Home Office licence in accordance with the Animals (UK Scientific Procedures) Act 1986 (Amendment Regulations 2012) following ethical review by Animal Welfare and Ethical Review Body (AWERB) at the University of Cambridge. All procedures were complied with guidelines set out by the International Association for the Study of Pain guidelines for the care and use of animals and were in accordance with the European Community Council Directive of 24 November 1986 (86/609/EEC). The right facial nerve of 8-week-old to 10-week-old male WT mice (C57BL/6, Harlan) was transected at its extracranial course near the stylomastoid foramen under fluothane (2%) anaesthesia with oxygen (1.5 l h^−1^). Before surgery, a subcutaneous injection of buprenorphine (Vetergesic; 0.1 mg kg^−1^) was administered to minimise pain and discomfort together with antibiotics (penicillin and streptomycin) to minimise potential infection.

### Sciatic nerve transection

All surgical procedures for sciatic nerve lesions were carried out by SF in accordance with the European Communities Council Directive of 22 September 2010 (2010/63/EU) regarding the use of animals in research, and was approved by the Ethics Committee of the Institute of Experimental Medicine, AS CR (Prague). Surgeries were performed on symptomatic 90 day old adult transgenic SOD1^G93A^ mice (B6SJL-Tg(SOD1*G93A)1Gur/J, Jackson Laboratories) and WT mice (B6SJLF1/J, Jackson Laboratories). Mice were anaesthetised with isofluorane (2%) inhalation (Abbot Laboratories) and in aseptic conditions a transversal cut was made on the skin 1 cm distal to the trochanter major in the right hindlimb. After a careful retraction of the femoral muscles via septum biceps femoris, a right sciatic nerve was exposed. A full sciatic nerve transection was made at the most proximal part of the nerve. Perioperatively mice received a subcutaneous injection of Rimadyl (Pfizer, 4.4 mg kg^−1^ in 0.1 ml of water for injections).

### Unlesioned mice

All procedures were carried out following the guidelines of the UCL-Institute of Neurology genetic manipulation and Ethic Committees and in accordance with the European Community Council Directive of 24 November 1986 (86/609/EEC). Animal work was carried out under license from the UK Home Office in accordance with the Animals (Scientific Procedures) Act 1986 (Amended Regulations 2012). For the collection of naïve spinal cords samples, 94 ± 1 day old adult transgenic SOD1^G93A^ mice (B6SJL-Tg(SOD1*G93A)1Gur/J, Jackson Laboratories) and WT mice (C56BL/6-SJL mixed background, Jackson Laboratories) were obtained and housed.

### Tissue Collection

According to the local and international ethical guidelines described above, the adult animals were killed by a lethal injection of phenobarbital (300 mg kg^−1^) or pentobarbital (65–200 mg kg^−1^) in humane conditions following brief anaesthesia by fluothane (2%) or isofluorane (2%) at different time points post-facial nerve axotomy (1, 7, 14, 28 days) or post-sciatic nerve transection (1 and 7 days) and at 94 ± 1 days of age for unlesioned mice. Axotomised or unlesioned WT or SOD1^G93A^ mice were either perfused with 4% PFA solution in PBS or fresh frozen samples were collected. For immunohistochemical analysis frozen lumbar spinal cord (L4–L6) or brainstem blocks were cut at 10 μm thickness using a cryostat (Leica). For obtaining 0.8 × 0.8 mm needle-punch FMN samples, a series of 300 μm thick coronal brainstem sections were cut. For cell culture purposes P1 mice pups were killed by the schedule 1 protocol according to the UK Home Office guidelines.

### Mouse cell cultures and purification

Cerebral cortices of P1 C57BL/6 WT mice (Harlan Olac & Charles River) or *Gfap-Cre* ^*+/+*^ */Stat3-loxP* ^*+/+*^mice (*Gfap-Stat3*-CKO) and thoracolumbar spinal cords of P1 C57BL/6 WT mice (Harlan) were prepared as described in previous studies^1, 69^. In general, this method establishes a purity of over 95% for astrocytes with <1% microglia. For our in vitro experiments, >98% pure astrocyte cultures were used. To obtain high purity astrocyte cultures for RNA sequencing, primary astrocyte cultures were obtained as described and then a further purification step was performed using Anti-GLAST (ACSA-1) MicroBead Kit (Miltenyi Biotec) according to the manufacturer’s protocols. Purified astrocytes were cultured for 6 weeks (mature astrocytes) in 10% fetal bovine serum, 1 mM GlutaMAX in Dulbecco’s modified Eagle medium (DMEM, Life Technologies) to provide cells for in vitro assays. Tissue for *Gfap-Stat3*-CKO astrocyte cultures were kindly donated by C. Zhao and R.J.M. Franklin, and was derived from mice originally generated by M. Sofroniew as described in Garcia et al. 2004. In CKO astrocytes the *Stat3* gene sequence encoding the tyrosine residue (*Tyr705*) crucial for STAT3 activation has been deleted. Motor neuron cultures from WT C57BL/6 E15 (Harlan) embryos were established and cultured using published protocols^70^ (>92% purity for motor neurons) with only minor modifications. Briefly, after isolation spinal cord dorsal roots and column were pulled off by tweezers and the remaining ventral part of the spinal cord was kept in HypernateE (Thermo Fisher Scientific) prior to dissociation, then Percoll (Sigma) was used as gradient for neuronal separation from glia. Survival assays were performed at 4 weeks after plating.

### ALS and control patient-derived iPSC-astrocytes

Human samples were obtained and handled according to the UK Home Office regulations and local guidelines in the laboratories. In this study, either three or four iPSC lines from healthy controls and two lines from patients carrying the SOD1^D90A^ mutation were used. This also included an isogenic pair of control and SOD1-mutant hiPSC-astrocyte lines (collaboratively provided by Dr S-C Zhang). Details of the iPSC lines used in this study can be found in Supplementary Table 1. For assays using hiPSC-astrocytes, at least three independent astrocyte cultures (*n* > 3) were used per group. Spinal astrocyte derivation from hiPSCs was adapted from our published protocol^42^. Briefly, after neural conversion (7 days in a chemically defined medium containing 1 μM Dorsomorphin (Millipore), 2 μM SB431542 (Tocris Bioscience) and 3 μM CHIR99021 (Miltenyi Biotec)) neural precursors were patterned for 7 days with 0.5 μM retinoic acid and 1 μM purmorphamine, followed by a 4-day treatment with 0.1 μM purmorphamine. After a propagation phase (>60 days) with 10 ng ml^−1^ FGF-2 (Peprotech) and were terminally differentiated to astrocytes in presence of BMP4 (10 ng ml^−1^, R&D) and LIF (10 ng ml^−1^, Sigma-Aldrich). Phenotypic characterisation of astrocytes included immunolabelling for GFAP, Aldh1L1, EAAT1 and EAAT2. Cultures used for in vitro assays were over 99% pure (Supplementary Fig. 6). For each iPSC line, astrocytes derived from a minimum of three independent neural conversions were analysed. All cell cultures were tested a minimum of fortnightly for mycoplasma using a PCR-based assay and there were no positive results throughout this experimental period.

### EphB1 and IL-6 treatment

As serum is known to induce astrocyte activation, before treatment primary mouse astrocytes were washed once with HBSS and subsequently serum-starved for a variable time (1–24 h) in Sato’s serum-free medium (Insulin 10 μg ml^−1^, transferrin 100 μg ml^−1^, bovine serum albumin 300 μg ml^−1^, putrescine 16 μg ml^−1^, thyroxine 400 ng ml^−1^, tri-iodo-thyronine 300 ng ml^−1^, progesterone 60 ng ml^−1^, sodium selenite 40 ng ml^−1^, 1 mM Glutamax in DMEM, low glucose, pyruvate, Life Technologies). After serum starvation, astrocytes were treated with either pre-clustered rat recombinant EphB1-Fc (1, 5, 10 µg ml^−1^, R&D Systems) or IL-6 (50 ng ml^−1^, R&D systems) in Sato’s medium. For all experiments clustered EphB1-Fc was used unless stated otherwise and was referred to as ‘EphB1’ in the text. Before treatment hiPSC-derived astrocytes were cultured for 72 h in absence of BMP4 and LIF and then treated with either human recombinant EphB1-Fc (10 µg ml^−1^, Gentaur) or IL-6 (50 ng ml^−1^, R&D systems). Clustering of EphB1-Fc was obtained by incubating EphB1 with a clustering antibody (goat anti-human IgG, Fc fragment specific, 1:10, Jackson ImmunoResearch) for 30 min at RT. At the appropriate time point after the stimulation, cells were further processed for immunocytochemistry, WB or RNA extraction.

### Cell transfection and knock-down by siRNA

Astrocytes were seeded on 24-well plates (10^4^ astrocytes per well) or 6-well plates (2.5 × 10^5^ astrocytes per well) or glass coverslips coated with poly-d-lysine (13 mm diameter, 10^4^ astrocytes per coverslip). The transfecting agent used was Lipofectamine 2000 (Life Technologies), using 0.5 µg of plasmid DNA for the luciferase assay or 50 pmol of ephrin-B1 siRNA or non-targeting RNA per well in a 24-well plate. For cortical astrocyte experiments ephrin-B1 siRNA-sequences were 5ʹ-AGGGUGACUCUGACGGCAA-3ʹ, 5ʹ-GGUUGGACACUGACGGACU-3ʹ, 5ʹ-CGCACUAUGAAGAUCGUUA-3ʹ, 5ʹ-GUGGAGAUCUUAAGCGGGU-3ʹ (Thermo Scientific Dharmacon, ON-TARGETplus SMARTpool, L-051210-01-0005) and for spinal cord astrocytes assays the siRNA sequence was 5ʹ-CGAUUACUACAUUACAUCA-3ʹ (Ambion Silencer Select). Non-targeting ‘scrambled’ RNA was used as control to reveal possible non-specific effects (Thermo Scientific Dharmacon, Non-targeting SMARTpool, D-001810-10-05, gift from Dr Jonathan Giley). After overnight incubation with the transfecting agent, the medium was replaced with fresh medium. Cells were analysed 1–3 days after transfection, depending on the downstream application.

### Immunolabelling

Frozen sections of mouse brainstems and mouse spinal cords were blocked in 10% normal goat serum (NGS) or 10% normal donkey serum as appropriate and permeabilised in 0.3% Triton X-100 (Sigma-Aldrich; in PBS) at RT for 30 min. They were then stained with primary antibodies in NGS (3%) and Triton X-100 (0.1% in PBS) at 4 °C overnight followed by species-specific secondary antibodies in PBS for 1 h and DAPI/Hoechst (100 ng ml^−1^) for 5–10 min at RT. For immunocytochemistry, cells on coverslips were fixed in 4% PFA (Sigma-Aldrich), then were blocked in 5% NGS or NDS in PBS, 0.1% Triton X-100. For STAT3 and pSTAT3 immunostaining, permeabilisation in cold methanol (−20 °C, 10 min) was performed. Primary antibody incubation was performed for 2 h at RT or overnight at 4°C in 2% NGS, 0.1% Triton X-100 in PBS and were diluted as follows: rabbit anti-Aldh1l1, 1:200 (Abcam), mouse anti-Aldh1l1, 1:100 (Millipore), rabbit anti-Cebpd, 1:200 (Abcam), goat anti-ChAT, 1:100 (Millipore), rabbit anti-EAAT1 1:200 (Antibodies online), rabbit anti-EAAT2 (Abcam), rabbit anti-EphB1, 1:100 (Santa Cruz), rabbit anti-ephrin-B1, 1:100 (Santa Cruz), rabbit anti-GFAP, 1:500 (DAKO), mouse monoclonal anti-GFAP, Cy3-conjugated, 1:500 (Sigma-Aldrich), mouse monoclonal anti-GFAP (Sigma-Aldrich), chicken anti-GFAP, 1:500 (Abcam), mouse monoclonal anti-NeuN, 1:200 (Millipore), rabbit anti-Phlda3, 1:200, (Lifespan Biosciences), rabbit monoclonal and mouse monoclonal anti-STAT3, 1:200 (Cell Signalling), rabbit monoclonal anti-pSTAT3 (Tyr705), 1:100 (Cell Signalling), rabbit anti-Trim30, 1:200 (Novus). This was followed by incubation with species-specific secondary antibodies for 1 h at RT. For F-actin staining, incubation with Alexa Fluor® 488 Phalloidin (Life Technologies, 1:300) was performed during secondary antibody incubation.

### Image analysis and processing

Images were taken either by fluorescent (Leica DM6000, ×20–63 objectives) or confocal microscopes (Leica TCS SPE, z-stack step: 1 μm, ×63 objective). Automated imaging of iPSC-derived astrocytes was carried out using the Opera Phenix High Content Screening system (Perkin Elmer, ×20 objective). Chemiluminescence on membranes was detected and imaged with the Alliance 4.7 CCD Image System (UVITEC). Camera exposure and gain has been kept the same while collecting images from each experiment. Cell counts were performed manually. All analysis was performed using unmodified images. For semi-automated analysis, either the Leica Application Suite software (Leica), Fiji (ImageJ) or Colombus (Perkin Elmer) software were used, which was also manually verified. To analyse intensity of nuclear STAT3, Cebpd and PHLDA3 IR IntDen measurements were carried out using a standard plugin in Fiji. For unbiased comparison across different experiments IntDen measurements were normalised to the cell-free background. For immunohistochemical analysis of tissue sections an internal control (e.g. unlesioned CL side) was used within the sample to normalise between replicate samples or experimental groups for unbiased comparison for manual cell counts. For illustration purposes we have followed the recommended guidelines. Images were minimally processed in Adobe Photoshop, which had been applied equally to samples that were directly compared (e.g. to internal controls) without affecting data presentation. This included changes in exposure and/or gamma parameters when clear views were compromised by interference with remaining excessive DAPI staining and PBS crystals on coverslips and histological slides. Pseudo-colours were obtained for images in Fiji for immunofluorescent staining using a far-red tagged secondary antibody to label ChAT-positive motor neuron in sections (cyan) or for DAPI staining (white) for better contrast. For illustration of WB images exposure was equally increased for the whole membrane at the original exposure. In cases where signal was low to embed into Adobe Illustrator without obscuration of visual data exposure/gamma parameters were adjusted equally across the image. WBs were cropped for focussed visualisation leaving a 6-band width. Lines were inserted to separate experimental samples that were either not loaded on gels exactly next to each other or were cut separately (Fig. 1f).

### Western blots

Immunoblotting was performed according to standard protocols (Life Technologies). Electrophoresis was run in 4–12% gradient NuPAGE Novex Bis-Tris Pre-Cast Gels (Life Technologies) and followed by protein transfer to a PDVF or nitrocellulose membrane (Life Technologies) according to standard protocols. Blocking was performed in PBS 1×, 0.2% Tween, 5% dry milk powder (Marvel). Membranes were stained with the primary and secondary antibodies diluted in the same blocking solution. Primary antibodies used were as follows: mouse monoclonal anti-β-actin, 1:20,000 (Abcam), rabbit anti-EphB1, 1:500 (Santa Cruz), rabbit anti-ephrin-B1, 1:1000 (Santa Cruz), mouse monoclonal anti-GFAP, 1:500 (Sigma-Aldrich), rabbit monoclonal anti-STAT3, 1:1000 (Cell Signalling), rabbit monoclonal anti-pSTAT3 (Tyr705), 1:1000 (Cell Signalling), rabbit anti-Trim30, 1:1000, (Novus). Detection was performed by exposing the membrane to the Amersham ECL Prime WB Detection Reagents (GE Healthcare).

### TMT labelling for mass spectometry

TMT labelling for MS (6 plex) was carried out according to the company’s protocol (ThermoScientific). For protein samples 1–2 × 10^6^ astrocytes were harvested in 8 M urea, 0.1% SDS in 50 mM TEAB (Triethyl ammonium bicarbonate) buffer supplemented with HALT protease and phosphatase inhibitors (ThermoFisher Scientific) and benzonase nuclease (Novagen). 100 μg protein samples at 1 μg μl^−1^ concentration in ultrapure water were reduced with 5 μl of 200 mM TCEP(Tris(2-carboxyethyl)phosphine) as per the manufacture’s instruction for the TMT kit (ThermoScientific). Samples were incubated for 1 h at RT to prevent urea derived carbamylation of lysine side chains, then were alkylated by addition of iodoacetamide (375 mM) in TEAB for 30 min before precipitation with 1 ml cold (−20°C) acetone and kept at −20°C overnight. Next, samples were centrifuged at 10,000 × *g*, at 10°C for 10 minutes and the pellet was briefly dried after the removing the supernatant before the digestion step in trypsin (2.5% in 100 mM TEAB). Labelling reaction was carried out by adding 0.8 mg of each TMT tag was dissolved in 41 μl acetonitrile and incubated on a shaker for 1 h at RT. The reaction was stopped by the addition of 8 μl 5% hydroxylamine and incubated at RT for 15 min. Samples were dried using refrigerated freeze-drier and then dissolved in 200 μl of 20 mM ammonium formate before loading 100 μl volume on to a reverse phase chromatography column (C18, Waters) for separating it into 32 fractions first. Finally, these were combined into 16 fractions and were freeze-dried before MS analysis.

### Proteomic analysis and data processing

Proteomic analysis has been carried out using an Orbitrap™ Fusion™ Lumos™ Tribrid™ mass spectrometer (Thermo Fisher Scientific) at the Cambridge Centre for Proteomics, using previously published parameters and methods^71^ employing synchronous precursor selection (SPS)–MS3. Briefly, TMT labelled and fractionated samples (~1 µg) were loaded onto Liquid chromatography-MS/MS analysis, using Orbitrap Fusion Lumos Mass Spectrometer coupled with a Dionex Ultimate 3000 nano-LC pump. An SPS–MS3 method was employed for the fragmentation of labelled peptide ions and subsequent fragment ions. Proteome Discoverer v2.1 (Thermo Fisher Scientific) and Mascot v2.6 (Matrix Science) were used to process raw data files. Data was aligned with the UniProt sequence database for *Homo sapiens*, in addition to using the common repository of proteins that typically contaminate shot proteomics experiments. Protein identification has allowed a tolerance of ±10 ppm and ±0.8 Da along with permission of up to two missed tryptic cleavages. Peptide-spectrum matches were re-scored for both ‘forward’ and ‘decoy’ searches by Mascot and the findings were filtered by peptide score of 20 and peptide confidence ‘high’. For accurate comparison data from control and SOD1-mutant hiPSC-astrocytes were normalised to the median of protein abundance values. Then candidate proteins were selected for data validation based on the RNA-seq and qPCR analysis, and the protein levels were presented as fold change (Supplementary Data File).

### Luciferase assay

To demonstrate STAT3-driven transcription activation astrocytes were transiently co-transfected with two reporter plasmids. The first one carried the cDNA for the Firefly Luciferase under the control of a STAT3-responsive promoter, and a second one included the Renilla Luciferase cDNA under a constitutively active promoter to normalise for the transfection efficiency. The following plasmids were used: pGL4.47[*luc2P*/SIE/Hygro] Vector (Promega) carrying five copies of the STAT3-inducible element (SIE) that drives transcription of the luciferase-reporter gene *luc2P* (*Photinus pyralis*, 2) pGL4.74[hRluc TK] Vector (Promega) that contains the *hRluc* luciferase-reporter gene under the control of a constitutive promoter (HSV-TK). Astrocytes (10^4^ cells per well in a MW24) were transfected with 500 ng of pGL4.47[*luc2P*/SIE/Hygro] and 50 ng of pGL4.74[hRluc TK]. After overnight incubation with the Lipofectamine-DNA complexes, the medium was replaced with fresh Sato’s medium for 24 h. Subsequently, cells were treated with either IL-6 or clustered EphB1 for further 24 h. At the end of the stimulation cells were rinsed in PBS and lysed in Glo Lysis Buffer (Promega). Subsequently Dual-Glo® Luciferase Reagent (Promega) and Dual-Glo® Stop & Glo® Reagent (Promega) were added to each sample, acting as substrate for the *luc2P* and Renilla luciferase, respectively. Luminescence was detected using the FLUOstar Omega plate reader (BMG Labtech).

### RNA extraction

For gene expression profiling mouse astrocytes were purified using the GLAST1 Microbead Kit (Miltenyi Biotec) as described above. Stimulation was performed as described above with the following modifications: one hour serum starvation in Sato’s medium, followed by 11 h of stimulation with either IL-6 (50 ng ml^−1^) or clustered EphB1 (5 μg ml^−1^). Non-treated astrocytes were kept in Sato’s medium. At the end of the treatment cells were washed with cold sterile PBS, collected and snap frozen. Total RNA was extracted using the RNeasy Plus Mini Kit (Qiagen) according to the manufacturer’s protocol.

### Reverse transcription and qPCR

Reverse transcription was performed using the Revert Aid First Strand cDNA Synthesis Kit (ThermoFisher Scientific) using 0.1–1 μg of total RNA. qPCR was performed using the Power SYBR Green Master Mix (ThermoFisher Scientific). Primers were chosen from the primePCR library (Bio-Rad) with a preference for intron-spanning primers, when available. A list of the primers used can be found in Supplementary Table 2. Gene expression data were analysed using the *ddCt* method using GAPDH as housekeeping gene.

### RNA sequencing

Library preparation was performed according to the Illumina TruSeq RNA Access library preparation kit as per the manufacturers instructions. Briefly, 100ng total RNA was first fragmented, cDNA is next generated using random priming during first and second strand synthesis and sequencing adaptors are ligated to the resulting double-stranded cDNA fragments. The coding regions of the transcriptome are then captured from this library using sequence-specific probes, then a final round of PCR amplification and second strand digestion occurs to create the final library. This strand-specific protocol was used for library preparation and samples were barcoded and multiplexed before sequencing on a HiSeq platform.

### RNA-seq and interactome analysis

A bioinformatic pipeline was constructed whereby the following steps were employed; fastQC, pre-processing, read alignment using Tophat2, htseq-count file generation and DESeq2 analysis. At least 80% of all reads aligned to the genome across all samples, of which a minimum of 60% were exonic. Approximately 90% strand specificity was confirmed. Counts were normalised using the DESeq2 variance stabilising transformation command prior to further analysis of differential expression and visualisation of results. Significance thresholds were set at FDR < 0.1 unless otherwise stated, and was determined using the procedure of Benjamini and Hochberg. Scatterplots and mean-centred heatmaps were generated in R. GO term analysis was performed using the GOSeq R package^72^. Transcription factors were defined by published recommendations^73^. Mouse and human STAT3 interactomes used to explore transcriptional signatures were generated in PathwayCommons (http://pathwaycommons.org), Biobase and IPA (Qiagen) platforms. Significantly induced mouse transcripts were analysed for their regulation by STAT3 using IPA, provided by Qiagen through the Hutchison/MRC Research Centre, Cambridge (www.ingenuity.com, 2017) and by the GO–Panther pathway analysis tool (http://pantherdb.org, 2017). Illustrations for STAT3 interactomes were generated in the Cytoscape platform, in which both the intensity of colour and the size of nodes representing different genes correlate with the degree of expression values.

### Statistical analysis

For in vivo experiments, the mouse subjects were not specifically randomised but were blinded for the observers. All quantified experiments for in vivo mouse models included both biological (*n* = 4 mice) and technical replicates (3–4 histological sections each at least). In vitro experiments using mouse astrocytes included three independent cultures from six different mice with technical replicates (coverslip cultures) for assays and for validation of gene expression profiles unless stated otherwise. The sample size was estimated from pilot and previously performed experiments^1^. For assays using hiPSC-astrocytes, at least three independent astrocyte cultures (*n* > 3) were used per group, which were converted from three healthy control or two ALS patients with the SOD1^D90A^ mutation (Supplementary Table 1). Data and graphs are presented as mean ± SEM, and ‘*n*’ values refer to the number of cells, cultures, tissue samples or animals analysed per group. GraphPad Prism 5, 6 and 7 (GraphPad Software) were used to generate graphs and to perform tests for distribution and statistical significance. Data were analysed using two-tailed, unpaired *t*-test for comparison of two groups, which was referred to as ‘*t*-test’ in the text. One-way or two-way analysis of variance was applied for comparison of multiple groups with Tukey’s post hoc test. Dunnett’s test was applied for comparison of means to the control group mean. Bonferroni correction was applied to examine apriori hypotheses for comparisons of specific pairs. The type of statistical tests with p and F values are also indicated in the figure legends. Statistical significance was accepted at *p*-values of  < 0.05. *p,**p, ***p indicate < 0.05, < 0.01, < 0.001, respectively. Non-significant *p*-values were labelled as “ns” in the text or in figures where relevant.

### Data availability

All transcriptomic data have been deposited in the GEO repository. The accession codes for new data on mouse and human iPSC-astrocytes presented are GSE102902, GSE102903. For further comparison of human iPSC, motor neuron and astrocyte differentiation states we also used our previously deposited data with accession numbers GSE98288, GSE99843. Experimental data relevant to the focused proteomic analysis is included in a supplementary file. Data is also available from the corresponding authors at request.

## Electronic supplementary material

Supplementary information

Description of Additional Supplementary Files

Supplementary Data 1
