# Supplementary material for: A neuroprotective astrocyte state is induced by neuronal signal EphB1 but fails in ALS models
Source: Nat Commun. 2017 Oct 27;8:1164. doi: 10.1038/s41467-017-01283-z (PMC5660125; doi:10.1038/s41467-017-01283-z)
Supplement: Supplementary file 1 — Supplementary information [file 41467_2017_1283_MOESM1_ESM.pdf]

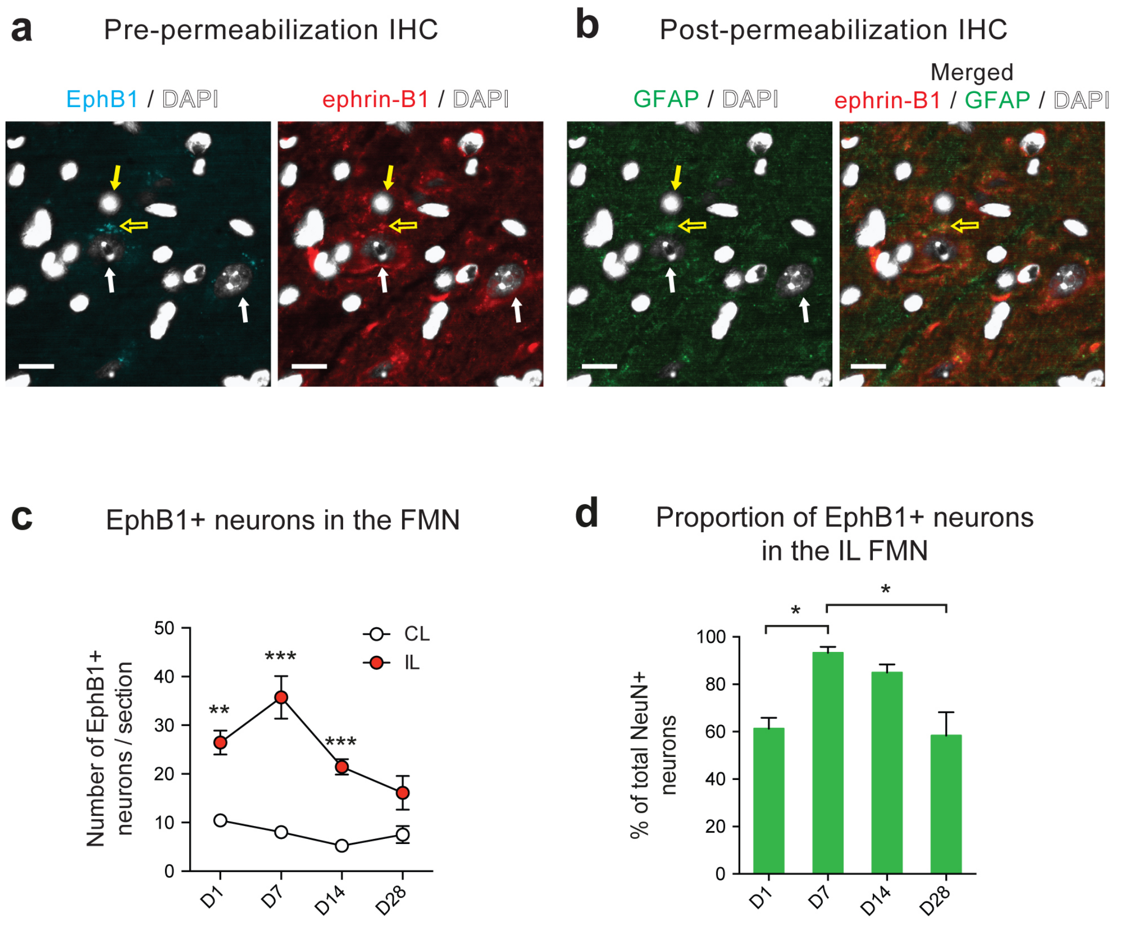

Supplementary Figure 1 | EphB1 expression in axotomized neurons in the facial motor nucleus. (**a**) EphB1 and ephrin-B1 immunostaining in the axotomized facial motor nucleus (FMN) without permeabilization of sections and (**b**) co-localizing ephrin-B1/GFAP IR following permeabilization with 0.1% Triton. (**c**) Graph showing average number of EphB1 positive neurons per section in both the IL and CL FMN post facial nerve axotomy (n = 3, 3, 3, 4 per timepoint, respectively; $^{**}p \leq 0.01$, $^{***}p \leq 0.001$, $F_{time} = 100.8$ and $F_{axotomy} = 7.7$; two-way ANOVA with Bonferroni comparison between IL and CL values). (**d**) Bar graph showing mean percentage of EphB1 positive neurons in the IL FMN over total NeuN labeled cells (n = 3 mice; $^{*}p \leq 0.05$, F = 6.25; one-way ANOVA with Tukey post-hoc test; see also Fig. 1). Data expressed as ± SEM. Scale bar: 30 μm.

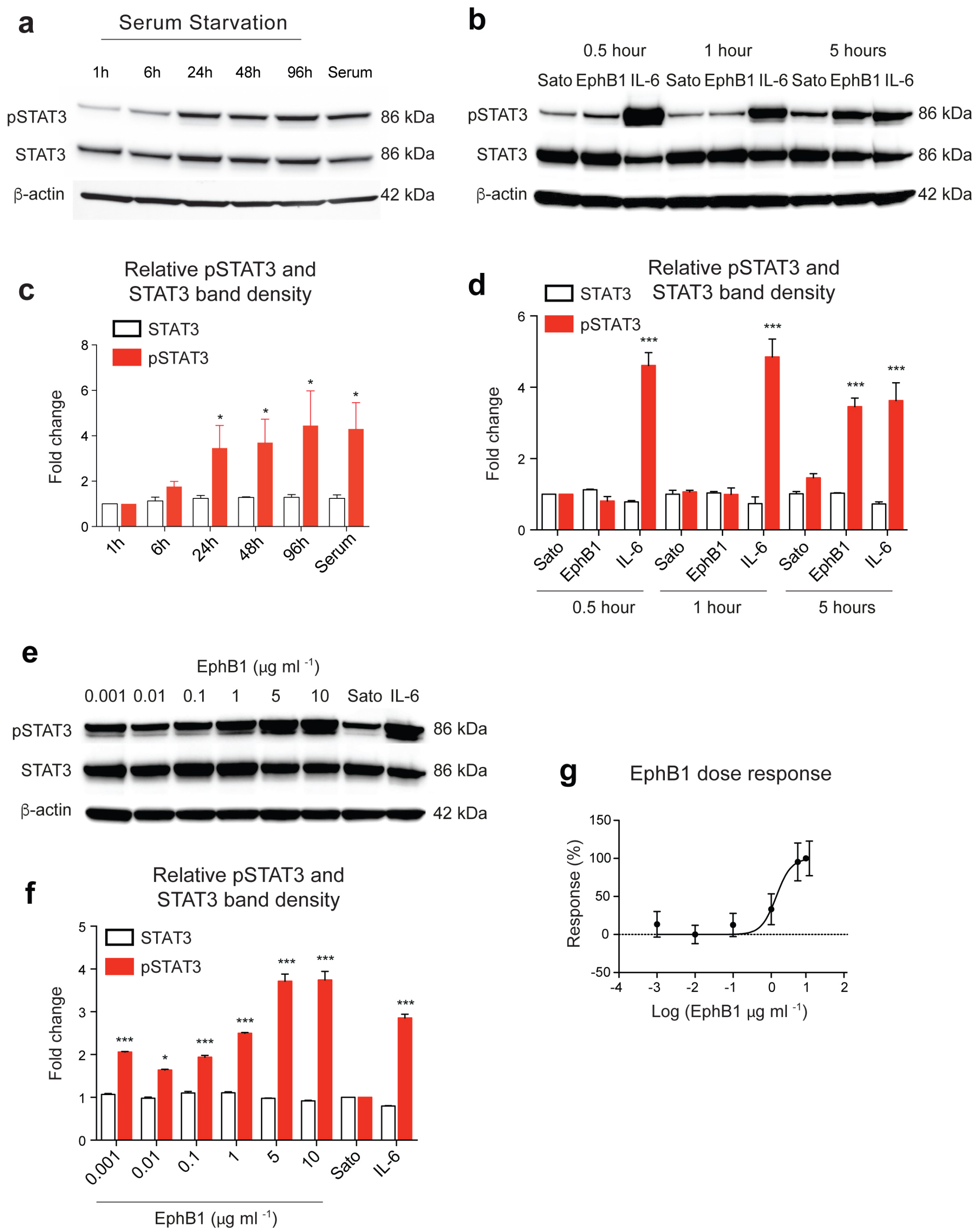

Supplementary Figure 2 | Optimization of EphB1 treatments for assessing STAT3 phosphorylation. (**a**) STAT3 phosphorylation response detected by western blotting to serum starvation at 1, 6, 24, 48 and 96 hrs in comparison with total STAT3 and β-actin IR and to the effect of serum. (**b**) Western blot shows STAT3 phosphorylation at 0.5h, 1h or 5 hours of treatment with either IL-6, or clustered EphB1 (5 µg ml^{-1}). (**c**,**d**) Graphs demonstrate relative band densities when compared to the 1-hour value for **a** and **c** and 0.5-hour value for **b** and **d** after normalization to the β-actin band for each time point. N = duplicate cultures of 6 mice; $*p = 0.044$, $*p = 0.029$, $*p = 0.015$, $*p = 0.033$, $F = 4.6$ for **c** and $***p \le 0.001$, $F = 120.8$ for **d**; one-way ANOVA with Dunnett's comparison. (**e**) Blots demonstrate STAT3 phosphorylation following a stimulation of 5 hours with clustered EphB1 at the following concentrations: 0.001, 0.01, 0.1, 1, 5, 10 µg ml^{-1}. Serum free medium (Sato) was used as negative control. (**f**) Bar graphs show relative band densities of pSTAT3 and total STAT3 to the control samples (Sato) after normalized to β-actin density. N = duplicate cultures from 6 mice; $***p \le 0.001$, $*p \le 0.05$, $F = 232.1$; one-way ANOVA with Dunnett's post-hoc test). (**g**) A dose-response curve was obtained by measuring levels of STAT3 phosphorylation by western blots. Data plotted is log of EphB1 concentration (µg ml^{-1}) against percentage of elicited response ($EC_{50} = 1.359$). Data expressed as mean of fold increase ± SEM. See also Suppl. Fig. 10 for western blots.

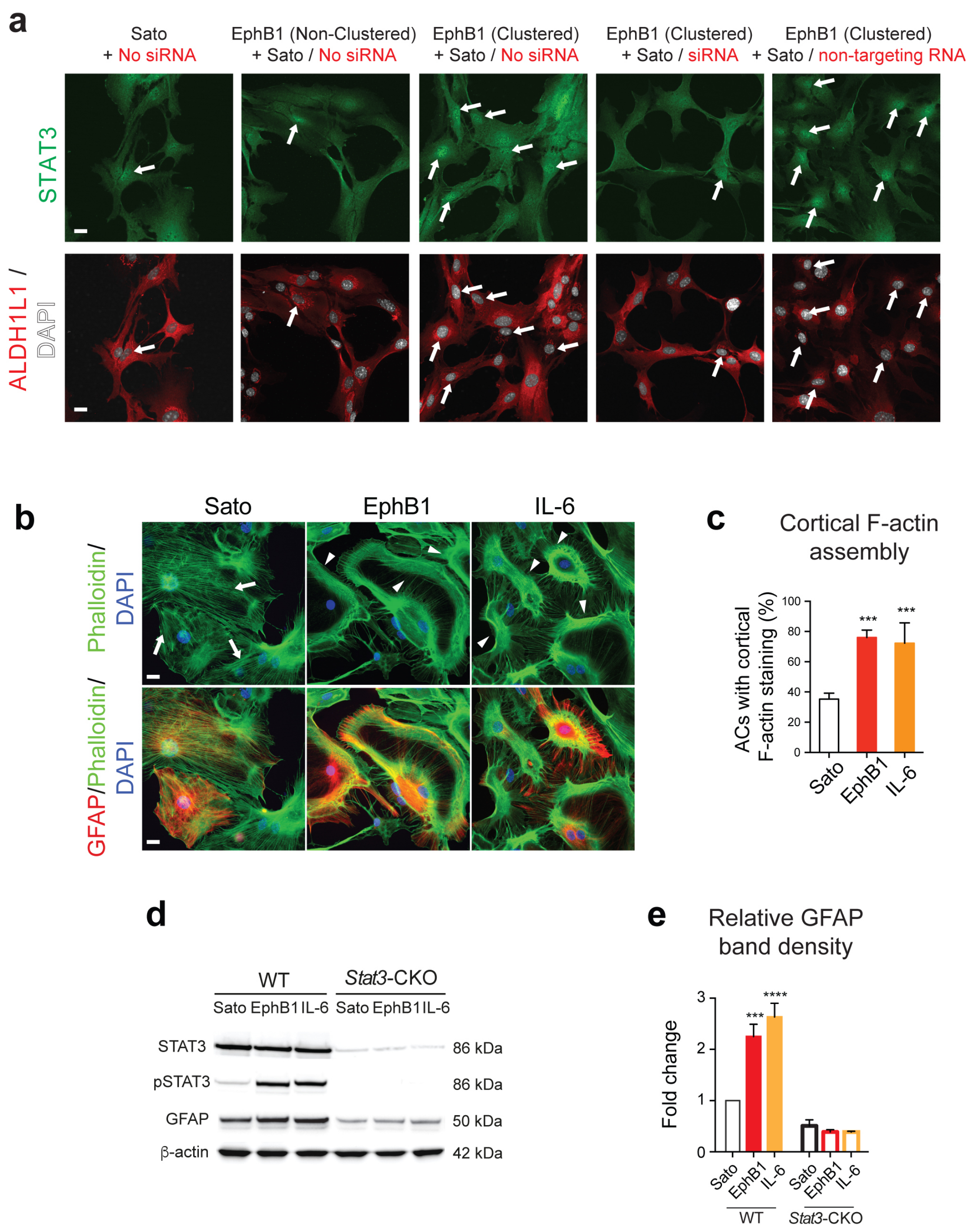

Supplementary Figure 3 | EphB1 induces STAT3 activation via ephrin-B1, and triggers STAT3-dependent reactive morphological transformation in astrocytes. (**a**) Immunofluorescence images of nSTAT3 and ALDH1L1/DAPI IR in corresponding spinal astrocytes (ACs) treated with non-clustered or clustered EphB1-Fc with or without ephrin-B1 siRNA or non-targeting RNA (also see **Fig. 3**). (**b**) Representative images showing EphB1 and IL-6-induced rearrangements of the actin cytoskeleton in cortical astrocytes. Phalloidin labelled F-actin is mostly organised in parallel fibres (*full arrows*) in Sato's medium. astrocytes stimulated with EphB1 or IL-6 display organized thick cortical actin rings with radial thinner fibres (*arrowheads*). (**c**) Bar graph shows the percentage of astrocytes bearing cortical thick actin bundles. N = 5, 5, 6 from 3 cultures of 6 mice; $***p \leq 0.001$, F = 31.89; one-way ANOVA with Bonferroni post-hoc test. (**d**) Western blot on whole cell lysates analysing the effects of EphB1 treatment on the expression levels of total STAT3, pSTAT3 and GFAP in both WT and STAT3-CKO astrocytes. (**e**) Bar graph show relative band density of GFAP to control bands (Sato) as fold change after normalization to β-actin band densities. N = 3 independent cultures; $***p = 0.0004$; $****p \leq 0.0001$, F = 68.9; one-way ANOVA with Dunnett's comparisons). Data are expressed as mean ± SEM. Scale bar: 20 μm.

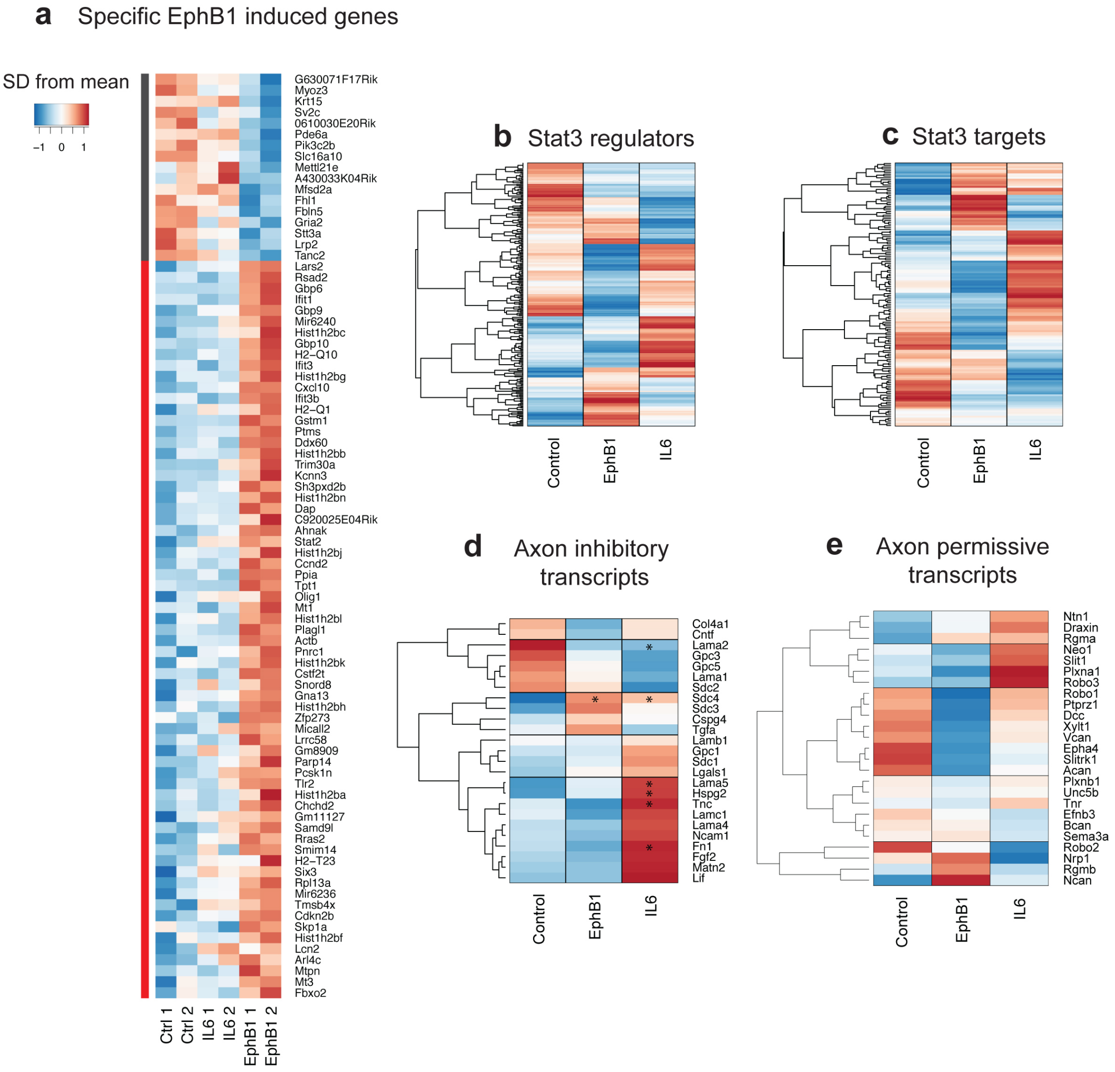

Supplementary Figure 4 | EphB1 and IL-6-induced mouse astrocytic transcriptome-wide signatures. (**a**) Heatmap showing specifically induced genes by EphB1 when compared to IL-6. Other heatmaps show comparisons between EphB1 or IL-6 with regard to induced genes related to (**b**) Stat3 regulators, (**c**) Stat3 targets, (**d**) axon growth inhibitory molecules and (**e**) axon growth permissive molecules (defined by published lists[3]). Gene expression data represent SD from mean of variance-stabilized values across rows.

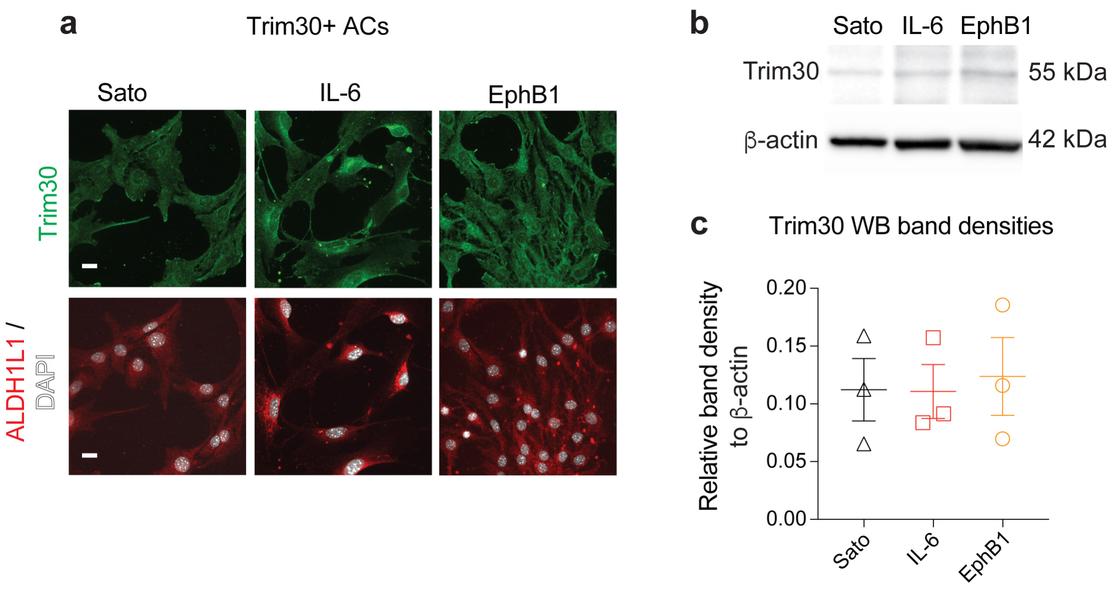

Supplementary Figure 5 | Additional characterization of EphB1 and IL-6 induced mouse astrocytic phenotypes. (**a**) Immunofluorescence demonstrating Trim30 IR in cultured ALDH1L1/DAPI positive astrocytes (ACs) exposed to IL-6 or EphB1 treatment or untreated. (**b**) Western blots for Trim30 and β-actin of the same astrocyte samples. (**c**) Dot plot graph represent WB band density measurements normalized to β-actin (n = 3 cultures of 6 mice; $p = 0.999$ and $p = 0.944$ for IL6 and EphB1 respectively, $F = 0.063$; one-way ANOVA with Bonferroni post-hoc test). Data expressed as ± SEM. Scale bar: 20 μm. See also Suppl. Fig. 10 for western blot.

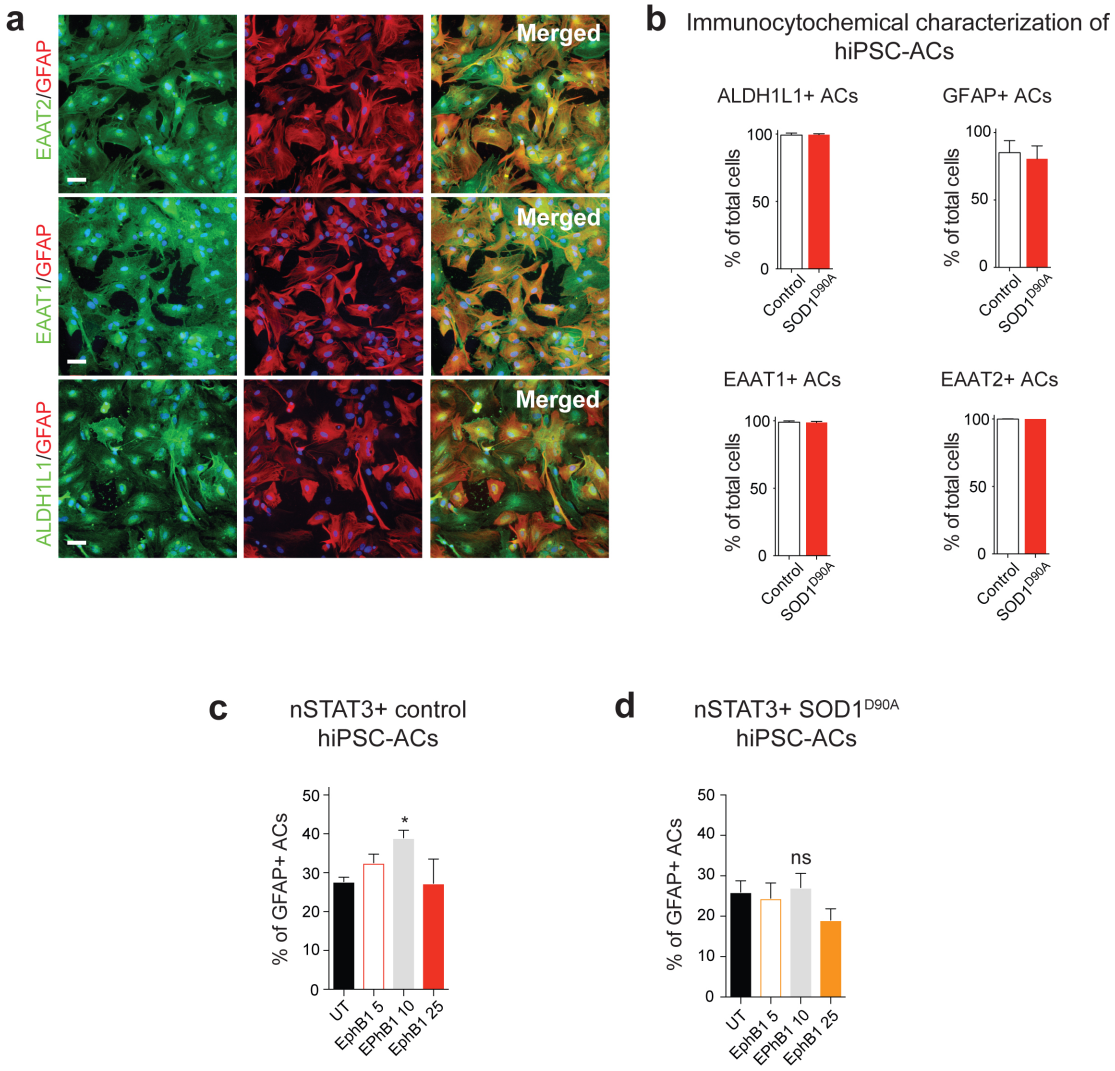

Supplementary Figure 6 | Characterization of hiPSC-astrocytes and their dose dependent response to EphB1. (**a**) Representative immunofluorescence images showing expression of astrocyte (AC) markers in hiPSC-astrocyte cultures, including ALDH1L1. GFAP, EAAT1 and EAAT2. (**b**) Bar graphs show the proportion of cells (%) positive for each marker over total number of cells identified by DAPI staining. (**c,d**) Dose dependent response of nSTAT3 IR in hiPSC-astrocytes to EphB1 treatment. Graphs represent the proportion of nSTAT3 positive cells amongst the total number of GFAP positive control (**c**) and in SOD1-mutant hiPSC-astrocytes (**d**). N = 6 cultures of 3 independently converted hiPSC-astrocytes from 2 healthy control and 1 ALS patient lines; $^*p = 0.046$, $F = 2.99$ for SOD1-mutant astrocytes and $F = 1.22$ for control astrocytes; one-way ANOVA with Dunnett's post-hoc test). Data is expressed as mean ± SEM. Scale bar: 40 μm.

Independent analysis of the isogenic (genetically corrected) control SOD1^{D90D} and mutant SOD1^{D90A} hiPSC-astrocytes

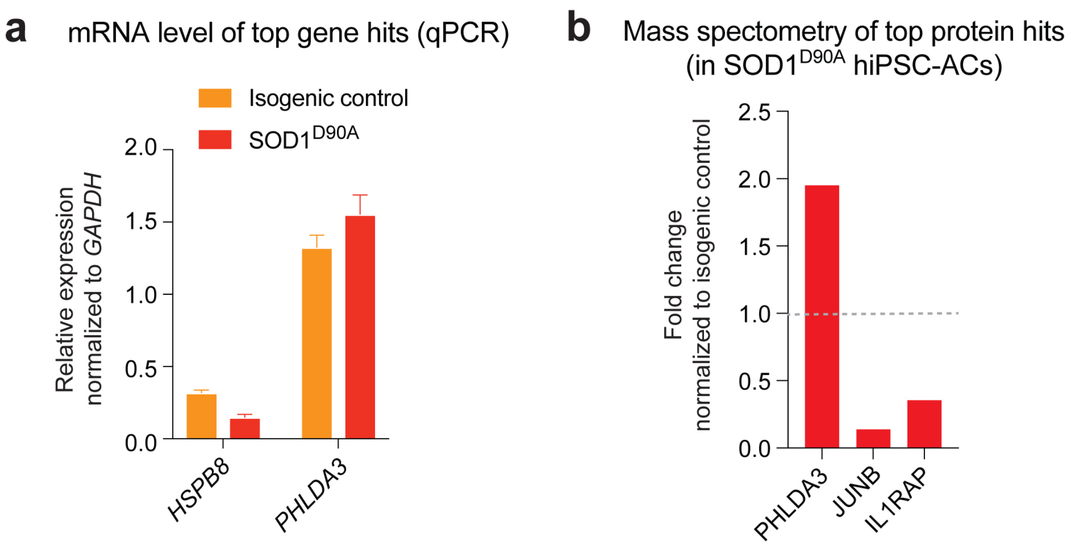

Supplementary Figure 7 | SOD1^{D90A}-mutant line and its isogenic control: independent verification of top findings in hiPSC-astrocytes by qPCR and mass spectrometry. (**a**) Graph indicates *HSPB8* and *PHLDA3* mRNA levels relative to *GAPDH* and expressed as fold-change (n = 2 independently converted astrocyte cultures). (**b**) Measurements of relative protein levels by mass spectrometry (MS) in SOD1 hiPSC-astrocytes (ACs) normalized to the levels for isogenic control astrocytes (n = one sample each of isogenic corrected and SOD1-mutant line with replicate labelling for MS).

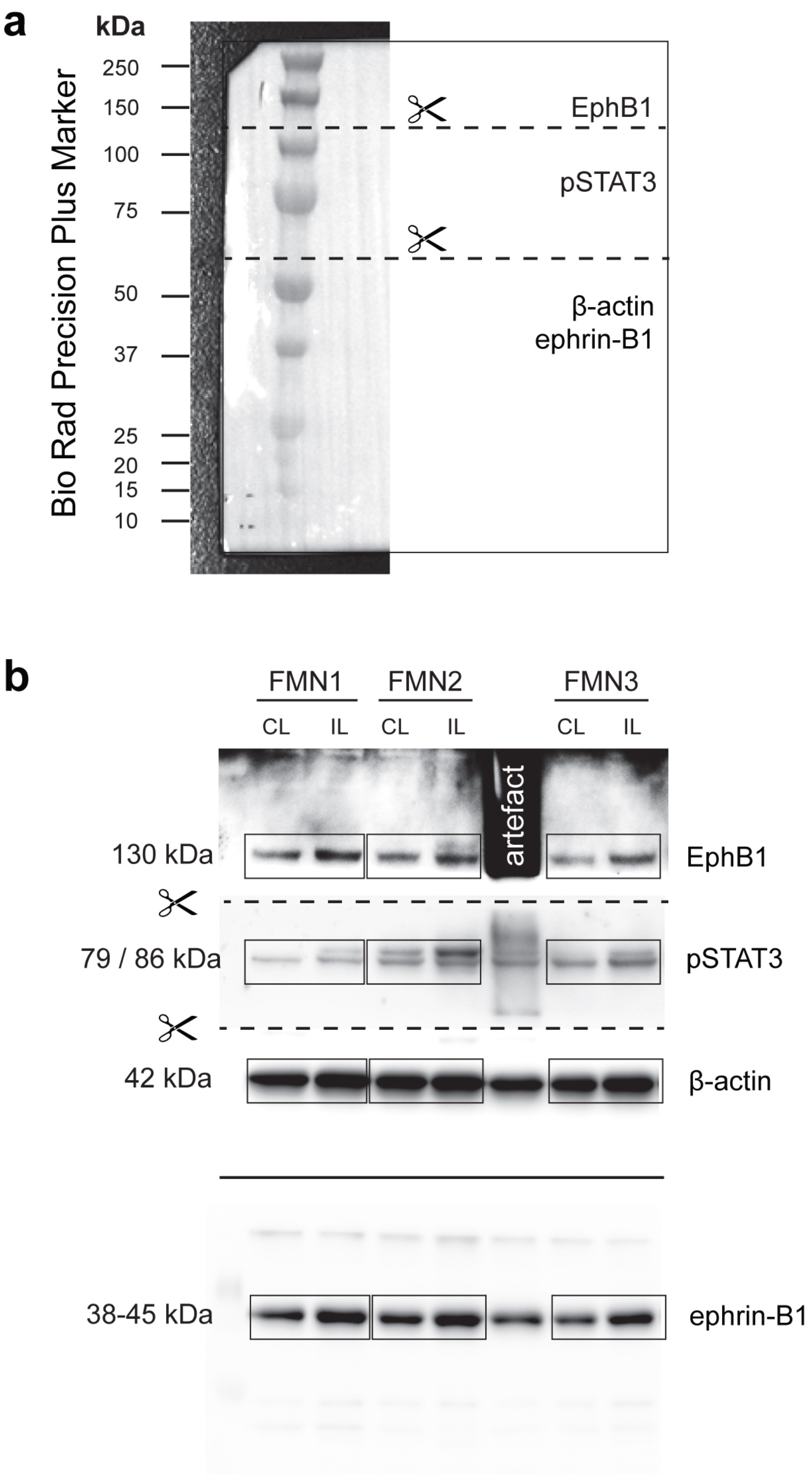

Supplementary Figure 8 | Whole western blots for Figure 1f**.** Membranes were cut guided by a marker (**a**) and/or stripped to enable simultaneous detection of multiple proteins in the same sample (**b**).

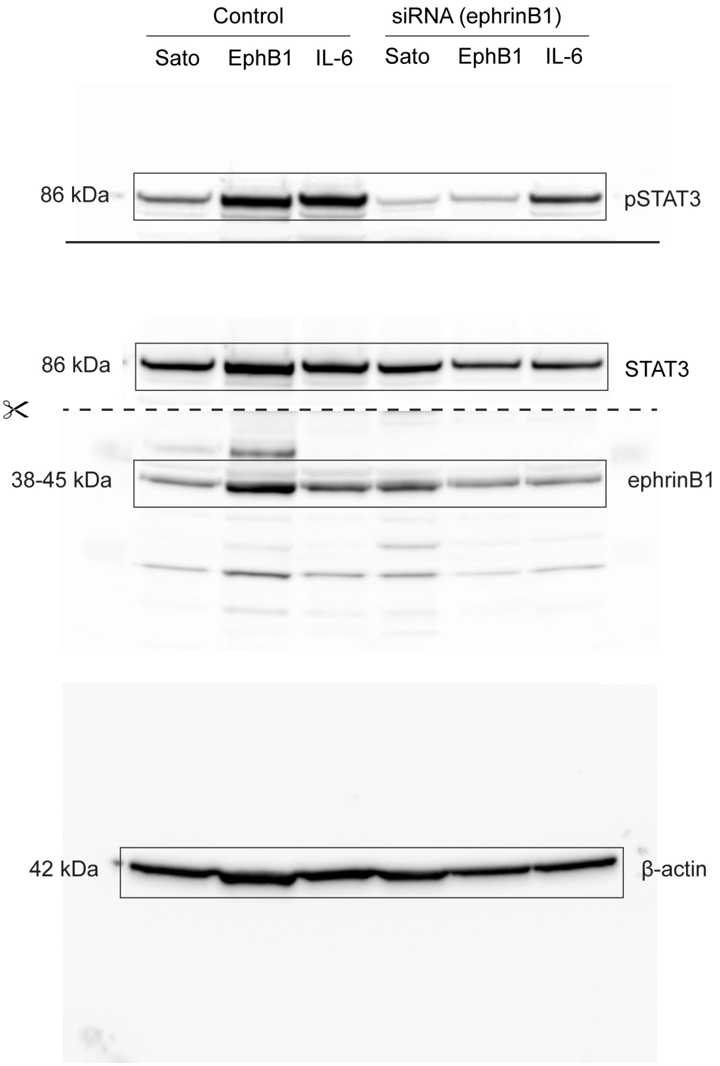

Supplementary Figure 9 | Whole western blots for Figure 2c**.**

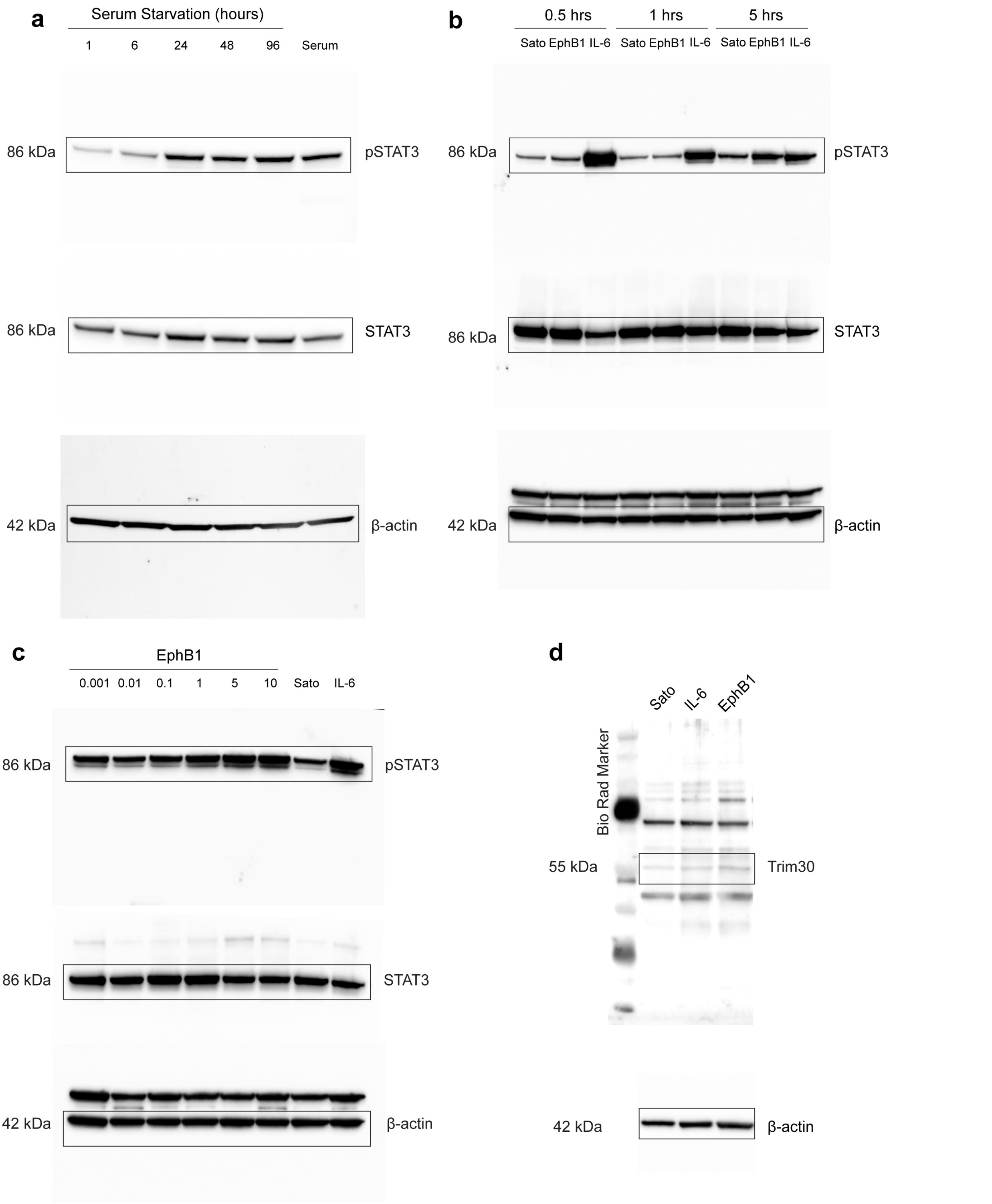

Supplementary Figure 10 | Whole western blots for Supplementary Figure 2a (**a**), 2b (**b**), 2e (**c**) and for Supplementary Figure 5b (**d**).

Supplementary Table 1 | Details of human iPSC lines used

**Line ID**	**Mutation**	**Age** (years)	**Gender**	**Source**
Control-1	None	78	Male	https://www.ncbi.nlm.nih.gov/pmc/articles/PMC4550814/
Control-2	None	64	Male	Corriell (ND41866*C)
Control-3	None	Unknown	Female	ThermoFisher Scietific (A18945)
SOD1-1	$SOD1^{D90A}$	70	Female	Coriell (ND35664)
SOD1-2	$SOD1^{D90A}$	50	Female	Su-Chun Zhang's lab
$SOD1^{D90D}$ Corrected isogenic control for SOD1-2	None	50	Female	Su-Chun Zhang's lab

Supplementary Table 2 | List of primers used for qPCR

Target	Species	Assay ID	Assay Info	Source
A2m	Mouse	qMmuCID0021890	Intron-spanning	Bio-Rad
Cebpd	Mouse	Mm_Cebpd_1_SG	Intron-spanning	Qiagen
Gapdh	Mouse	qMmuCID0027497	Exonic	Bio-Rad
Kcnn3	Mouse	qMmuCID0011935	Intron-spanning	Bio-Rad
Mt1	Mouse	qMmuCID0003677	Exonic	Bio-Rad
Trim30a	Mouse	qMmuCID0007007	Intron-spanning	Bio-Rad
CEBPD	Human	qHsaCED0002556	Exonic	Bio-Rad
GAPDH	Human	qHsaCED0038674	Exonic	Bio-Rad
HSPB8	Human	qHsaCID0018781	Intron-spanning	Bio-Rad
KCNJ10	Human	qHsaCID0013161	Intron-spanning	Bio-Rad
LRP2	Human	qHsaCID0012030	Intron-spanning	Bio-Rad
PHLDA3	Human	qHsaCID0022631	Intron-spanning	Bio-Rad
