# Supplementary material for: A neuroprotective astrocyte state is induced by neuronal signal EphB1 but fails in ALS models
Source: Nat Commun. 2017 Oct 27;8:1164. doi: 10.1038/s41467-017-01283-z (PMC5660125; doi:10.1038/s41467-017-01283-z)
Supplement: Supplementary file 2 — Description of Additional Supplementary Files [file 41467_2017_1283_MOESM2_ESM.pdf]

Description of Additional Supplementary Files

File Name: Supplementary Data 1
Description: TMT-Mass Spectrometry Proteomics - Increased protein levels in SOD1D90A versus control hiPSC-astrocytes (>1.5 fold)
